# Warming transforms the western Arctic Ocean into a hub of drifting matter

**DOI:** 10.1038/s41467-026-74439-5

**Published:** 2026-06-16

**Authors:** Kou Wang, Caili Liu, Qi Shu, Claudia Wekerle, Caixia Wang, Qiang Wang

**Affiliations:** 1 State Key Laboratory of Physical Oceanography, Ocean University of China, Qingdao, China; 2 Alfred Wegener Institute, Helmholtz Centre for Polar and Marine Research, Bremerhaven, Germany; 3 First Institute of Oceanography and Key Laboratory of Marine Science and Numerical Modeling, Ministry of Natural Resources, Qingdao, China; 4 Shandong Key Laboratory of Marine Science and Numerical Modeling, Qingdao, China

**Keywords:** Physical oceanography, Physical oceanography

## Abstract

The Arctic Ocean is increasingly stressed by anthropogenic pollution and rapid environmental change. River discharge plays a crucial role in this transition by delivering freshwater, nutrients, carbon, and contaminants to the ocean. Yet how river-borne materials will spread through the Arctic under future climate warming remains unclear. Here we show that climate warming accelerates and expands the dispersal of Arctic river waters and drifting materials through multi-scale changes in ocean circulation linked to sea-ice decline and reduced upper-ocean density. Stronger ocean eddy activity and altered wind-driven circulation transform the Beaufort Gyre from a predominantly regional reservoir into a pan-Arctic convergence zone that efficiently accumulates river-derived materials from Siberia. Meanwhile, intensified boundary currents and Transpolar Drift accelerate the export of Siberian discharge toward the North Atlantic. Together, these circulation changes greatly increase cross-basin connectivity, with broad implications for marine ecosystems in the northern high-latitude oceans and for Arctic coastal communities.

## Abstract (web summary)

Climate warming is transforming the western Arctic Ocean into a major accumulation zone of drifting materials from across the Arctic while accelerating connections between Siberian shelves and the North Atlantic.

## Introduction

The Arctic Ocean exerts a disproportionate influence on Earth’s climate system relative to its size. It regulates freshwater received from rivers, precipitation and melting ice, and modulates global circulation and climate through the export of freshwater to dense water formation regions in the North Atlantic^1^. The freshwater maintains the Arctic halocline, insulating the underlying warm Atlantic Water and thus sustaining Arctic sea ice^2^. Each year, Arctic rivers deliver thousands of cubic kilometers of freshwater, over 10% of global river discharge, into less than 1% of the global ocean volume^3^. These rivers also transport large amounts of terrigenous nutrients, carbon, and pollutants from high-latitude watersheds^4–7^.

River inputs profoundly influence the Arctic marine ecosystem^8^. Fed by terrigenous organic matter, the Arctic surface layer contains the highest concentration of dissolved organic carbon in the global ocean, strongly affecting marine ecosystem functioning^5,9,10^. Permafrost thaw, coastal erosion and enhanced river runoff are increasing terrigenous fluxes of nutrients to the Arctic Ocean^4,11–13^. Under future warming, these fluxes are projected to weaken the Arctic biological carbon pump and intensify coastal carbon outgassing, thus reducing the oceanic carbon sink^14,15^.

The world’s oceans are facing a growing burden of plastic pollution^16–20^, and even the remote Arctic Ocean is not exempt^7,21^. Plastic pollutants harm ocean wildlife through ingestion and entanglement^22,23^. They also contain toxins and absorb other pollutants, thereby contaminating food webs and posing risks to human health^24,25^. River discharge is one of the major pathways by which plastics enter the global ocean^26,27^. In the Arctic, while a large fraction of plastic pollutants originates from atmospheric transport and Atlantic inflow^28,29^, river discharge remains an important source^7,30^. Moreover, hazardous substances stored in permafrost could be released into rivers as permafrost thaws, posing additional risks^31,32^.

Understanding the evolving fate of Arctic river discharge is therefore critical. The circulation and residence timescales of river runoff exhibit interannual to decadal variability linked to shifts in atmospheric circulation regimes^33,34^. The Transpolar Drift serves as a main conduit transporting Siberian matter toward the central Arctic and subarctic seas^35,36^, whereas the Beaufort Gyre acts as a major Arctic freshwater reservoir maintained by Ekman convergence driven by anticyclonic winds^37–39^. Under the present conditions, the Beaufort Gyre primarily accumulates freshwater from the Mackenzie River, sea ice melt and low-salinity Pacific inflow^37^. Although some Siberian discharge may enter the Beaufort Gyre under certain wind conditions^40^, its contribution is much smaller than that of the Mackenzie River^37,41^. Consistent with limited connectivity across the Transpolar Drift, floating particles of European origin show minimal concentration in the Beaufort Gyre^42^, and observed plastic pollution levels are lower in the western Arctic than in the Eurasian sector^7^.

The Arctic Ocean is undergoing coupled physical and biogeochemical transformations. Permafrost thaw, expanding industrial activity, and opening of new shipping routes and ports are increasing terrigenous and anthropogenic inputs^11,31,43–45^, while declining ice cover enhances oceanic circulation by strengthening air-sea momentum transfer and reducing ocean surface dissipation^46–48^. Yet, how these circulation changes reshape the fate of Arctic river inputs remains poorly understood.

Here, we investigate the dispersal of Arctic river discharge under a warming climate using eddy-permitting numerical simulations with passive tracers representing inputs from major Arctic rivers, combined with Lagrangian surface particle tracking. We show that the western Arctic Ocean emerges as a hub of drifting materials from both Eurasian and North American sources. Enhanced cross-basin connectivity promotes the accumulation of discharge from the remote Siberian rivers into the western Arctic, with broad implications for marine ecosystems and Indigenous communities.

## Results

### Faster dispersal of river discharge in a warming climate

**Fig. 1:**
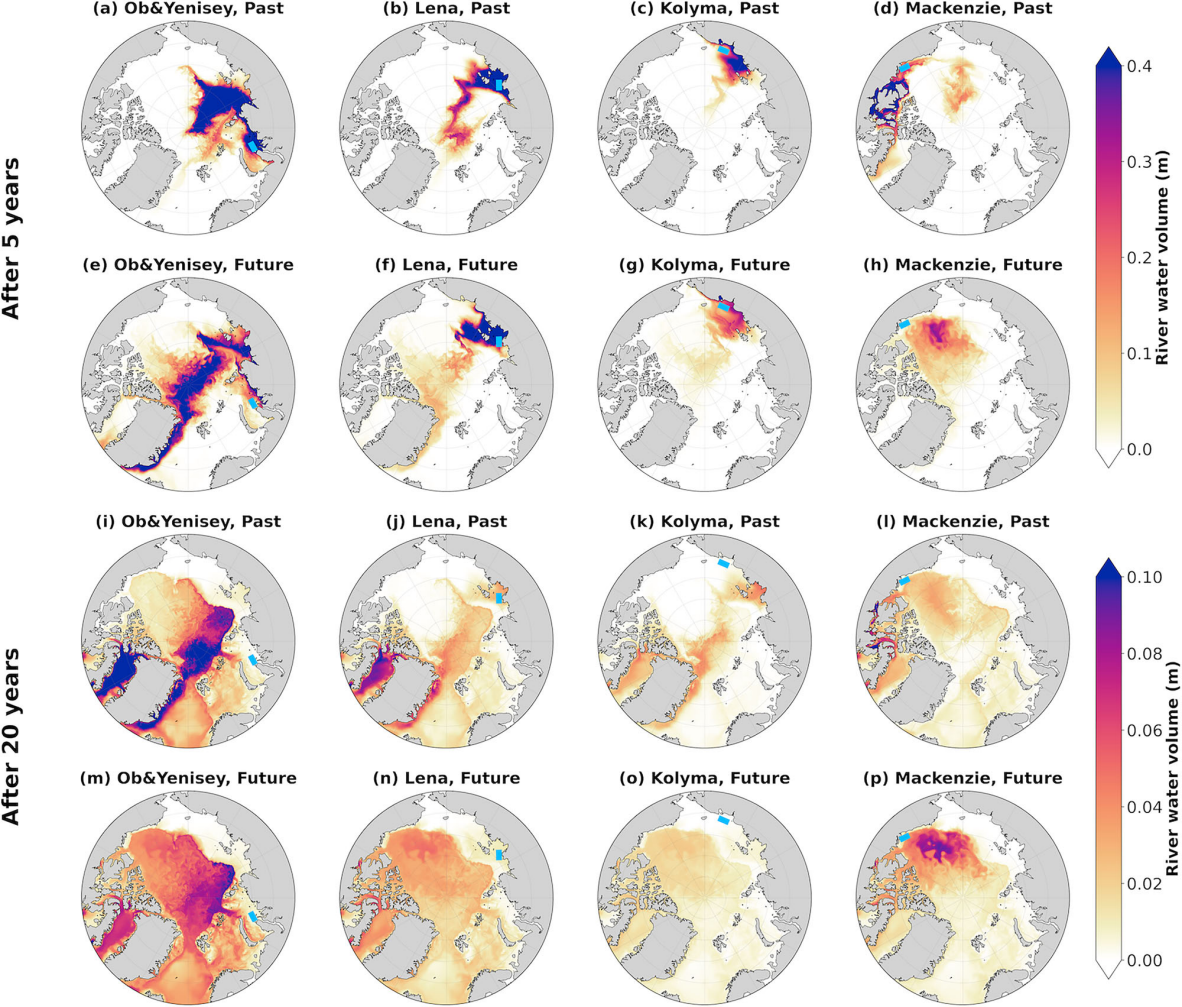
Dispersal of river discharge under different climate conditions. **a**–**d** Spatial distribution of vertically integrated river runoff content after entering the ocean for five years in the historical period: (**a**) Ob–Yenisey rivers, (**b**) Lena River, (**c**) Kolyma River, and (**d**) Mackenzie River. **e**–**h** The same as (**a**–**d**), but for the future period. **i**–**p** The same as (**a**–**h**), but after entering the ocean for 20 years. Temporal mean values averaged over the last month of the respective years are shown. The river mouths of the major rivers in different shelf seas are denoted by blue bars.

To quantify the dispersal of Arctic river discharge, we implemented online passive tracers in our model simulations. These tracers were initialized at zero and supplied by the freshwater flux associated with river runoff (see “Methods”). They were simulated for the historical period (1985–2004) and the future scenario (2071–2090, CMIP6 SSP585) separately. The river runoff forcing for the passive tracers was applied only during the first simulation year, allowing the subsequent evolution to reveal the dispersal of the riverine water initially introduced into the ocean. The evolution of the riverine water plumes over the first five years indicates a markedly accelerated dispersal of river discharge under future warming conditions (Fig. 1a–h).

**Fig. 2:**
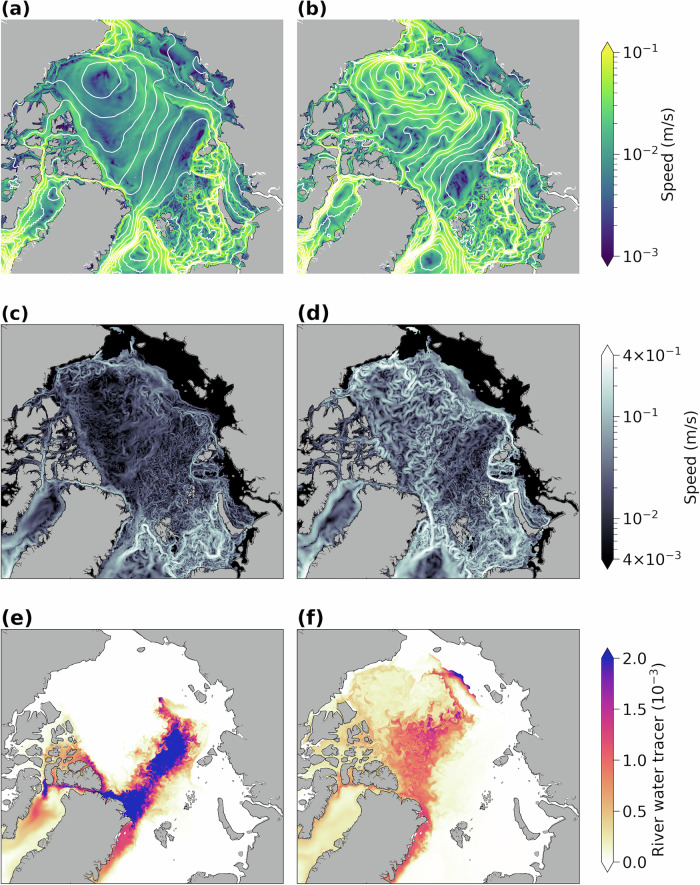
Strengthened cross-basin connectivity associated with multi-scale circulation changes under climate warming. **a**, **b** Mean ocean currents in the upper 100 m, averaged over (**a**) 1985–2014 and (**b**) 2071–2100. Color shading indicates current speed magnitude, while contour lines show sea surface height at 5 cm intervals, illustrating the directions of surface geostrophic currents. **c**, **d** Snapshots of ocean current speeds at 50 m depth at the end of (**c**) 1994 and (**d**) 2080, representing the midpoints of the tracer simulations. **e**, **f** Corresponding snapshots of the Lena River tracer at 50 m depth at the end of (**e**) 1994 and (**f**) 2080, coinciding with the time shown in (**c**, **d**).

In the historical period, most of the Ob–Yenisey riverine water does not reach the North Pole within five years after entering the ocean (Fig. 1a). In contrast, in the future scenario, the Ob–Yenisey discharge is advected more rapidly, with a large portion carried by the Transpolar Drift to the central Arctic, and some already exiting the Arctic Ocean after five years (Fig. 1e). This reflects an acceleration of the Arctic shelfbreak current and Transpolar Drift (Fig. 2a, b). The Lena plume still occupies the eastern Laptev shelf after five years in both climate scenarios (Fig. 1b, f). However, only in the future scenario does part of the Lena riverine water reach the east Greenland shelf by the end of the fifth year (Fig. 1b, f). The Kolyma discharge exhibits a relatively longer residence time over the shelf than the other Arctic rivers (Fig. 1c, g), but it similarly shows faster advection in the Arctic basin in the future scenario. For the Mackenzie River, part of its discharge enters the anticyclonic Beaufort Gyre circulation, while another portion exits the Arctic Ocean through the Canadian Arctic Archipelago straits (Fig. 1d). The partitioning between these two pathways depends on the strength of the anticyclonic gyre circulation^34^. In a warming climate, consistent with the strengthening of the Beaufort Gyre (Fig. 2a, b), most of the Mackenzie River discharge follows the anticyclonic pathway and accumulates in the Canada Basin during the first five years (Fig. 1h).

The absolute values of river water content shown in Fig. 1 are influenced by both ocean circulation and the amount of river discharge, which increases under a warming climate. By comparing particle tracking experiments in which the number of particles either accounts for the increase in river runoff or remains unchanged (see “Methods”), we find that the quantitative differences between these experiments are much smaller than those between the two periods considered (Supplementary Figs. 1 and 2). This suggests that changes in ocean circulation mainly drive the differences in the spatial distribution of river waters, particularly their faster dispersal under future climate.

**Fig. 3:**
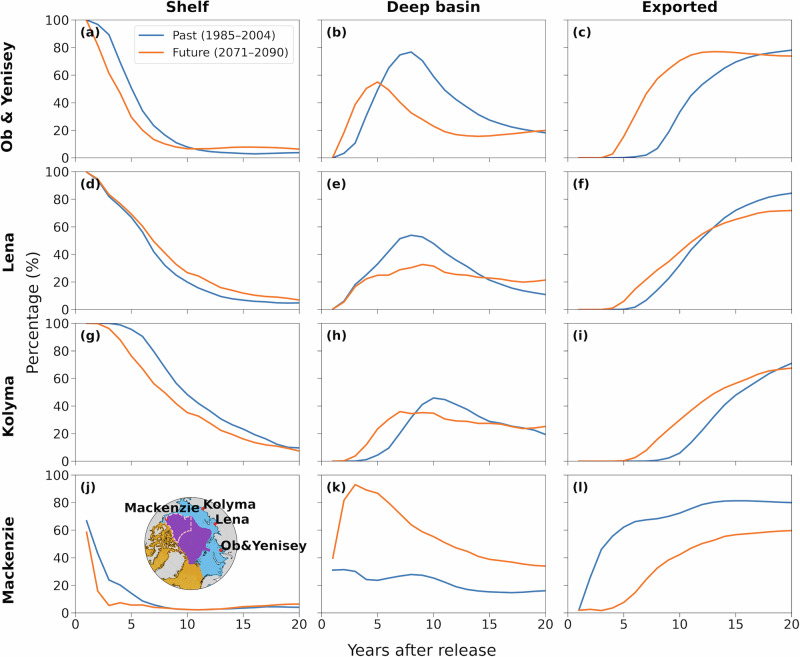
Accelerated advection of riverine matter in a warming climate. **a**–**c** Time series of the proportion of Ob–Yenisey river runoff (**a**) remaining over continental shelves, (**b**) located in the Arctic deep basin, and (**c**) exported out of the Arctic Ocean. **d**–**f** The same as (**a**–**c**), but for the Lena River runoff. **g**–**i** The same as (**a**–**c**), but for the Kolyma River runoff. **j**–**l** The same as (**a**–**c**), but for the Mackenzie River runoff. The historical period (1985–2004) is compared with the future period (2071–2090). In the inset in (**j**), the colors distinguish the three regions used in this figure, and the dashed lines indicate the Canada Basin.

Time series of river discharge fractions in different regions further confirm faster dispersal under climate warming (Fig. 3). By the end of the fifth year, shelf retention for the Ob–Yenisey plume drops from 65% historically to 30% in the future (Fig. 3a). The reduced residence time on the shelf leads to an earlier increase in the basin fraction (Fig. 3b). However, due to faster Transpolar Drift in the future scenario, the fraction of Ob–Yenisey discharge in the deep-basin area becomes lower than in the historical simulation after the fifth year, reflecting its more rapid export from the Arctic (Fig. 3b, c). It takes less than four years for the discharge to reach the Arctic export gateways in the future, in contrast to more than six years historically (Fig. 3c). Ten years after release, the fraction of Ob–Yenisey discharge exported from the Arctic increases from about 30% in the historical period to roughly 70% in the future (Fig. 3c). A portion of the exported Ob–Yenisey water entrains into the Atlantic Water and reenters the Arctic after about ten years, leading to a small rebound in its fraction in the Arctic, which is more obvious in the future due to faster Arctic export (Fig. 3a, b).

In contrast to the Ob–Yenisey plume, the shelf retention of the Lena plume is slightly larger in the future (Fig. 3d). This is associated with an eastward shift in the location where the Lena discharge leaves the shelf (Fig. 1b, f). However, once the Lena discharge enters the Transpolar Drift, it is exported from the Arctic Ocean more rapidly (Fig. 3e, f). The Kolyma discharge exhibits a response similar to that of the Ob–Yenisey, with accelerated export from the shelf to the deep basin and from the deep basin to the subarctic seas (Fig. 3g–i). Ten years after release, the fractions of Lena and Kolyma discharge exported from the Arctic are approximately 30% and 10%, respectively, in the historical period, increasing to over 40% and 30% in the future (Fig. 3f, i). The Mackenzie River discharge is projected to remain in the Arctic Ocean for a longer period in the future (Fig. 3j–l), with the fraction exported during ten years decreasing from 70% historically to 40% under the future condition (Fig. 3l).

### Strengthened accumulation of river discharge in the Canada Basin

Although the export of Siberian discharge via the Transpolar Drift to the subpolar North Atlantic is projected to accelerate in a warming climate, the penetration of Siberian river discharge into the western Arctic will also increase (Fig. 1i–p). In the historical period, the amount of Ob–Yenisey discharge in the Canada Basin is relatively small after 20 years, while the contributions from the Lena and Kolyma rivers are negligible (Fig. 1i–k). In the future period, the discharge from all the Siberian rivers exhibits a marked increase in volume in the Canada Basin (Fig. 1m–o). The Lena and Kolyma discharge even concentrates in the Canada Basin after 20 years (Fig. 1n, o). The river water returning to the Arctic after circulating through the Nordic Seas and subpolar North Atlantic increases the concentration of the Siberian discharge in the Barents Sea and Eurasian Basin. Surface particle tracking experiments, in which the reentry of particles to the Arctic Ocean was disabled, confirm that the majority of riverine particles remaining inside the Arctic after 20 years are located in the Canada Basin (Supplementary Fig. 1).

Among the rivers considered, the Ob–Yenisey discharge shows the largest changes in vertical distribution in the Canada Basin (Supplementary Fig. 3). Ob–Yenisey runoff mixes with denser Atlantic Water on the shelves^49,50^, allowing it to penetrate into the Canada Basin at greater depths. Under climate warming, the Ob–Yenisey discharge is located closer to the surface (Supplementary Fig. 3a, e), consistent with a reduction in shelf water density resulting from warming and freshening ^51^.

**Fig. 4:**
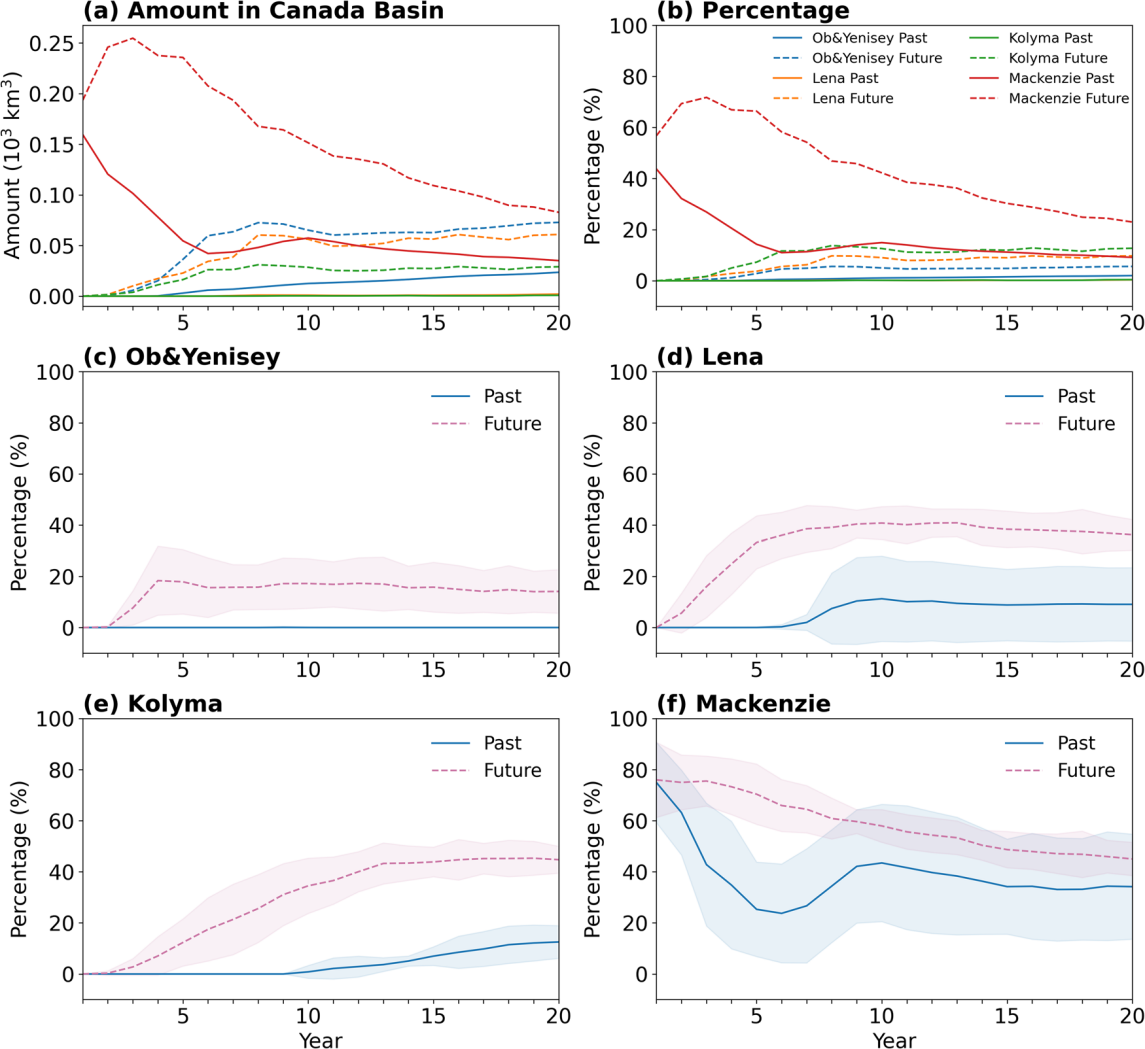
Increasing intrusion of riverine matter into the Canada Basin in a warming climate. **a** Time series of the volume of river runoff in the Canada Basin. **b** Time series of the proportion of river runoff in the Canada Basin relative to the total runoff from the respective river. **c**–**f** Time series of the proportion of surface particles in the Canada Basin relative to the total particles released from the respective river: (**c**) Ob–Yenisey, (**d**) Lena, (**e**) Kolyma, and (**f**) Mackenzie rivers. The shading areas indicate one standard deviation of 11 particle tracking experiments.

**Fig. 5:**
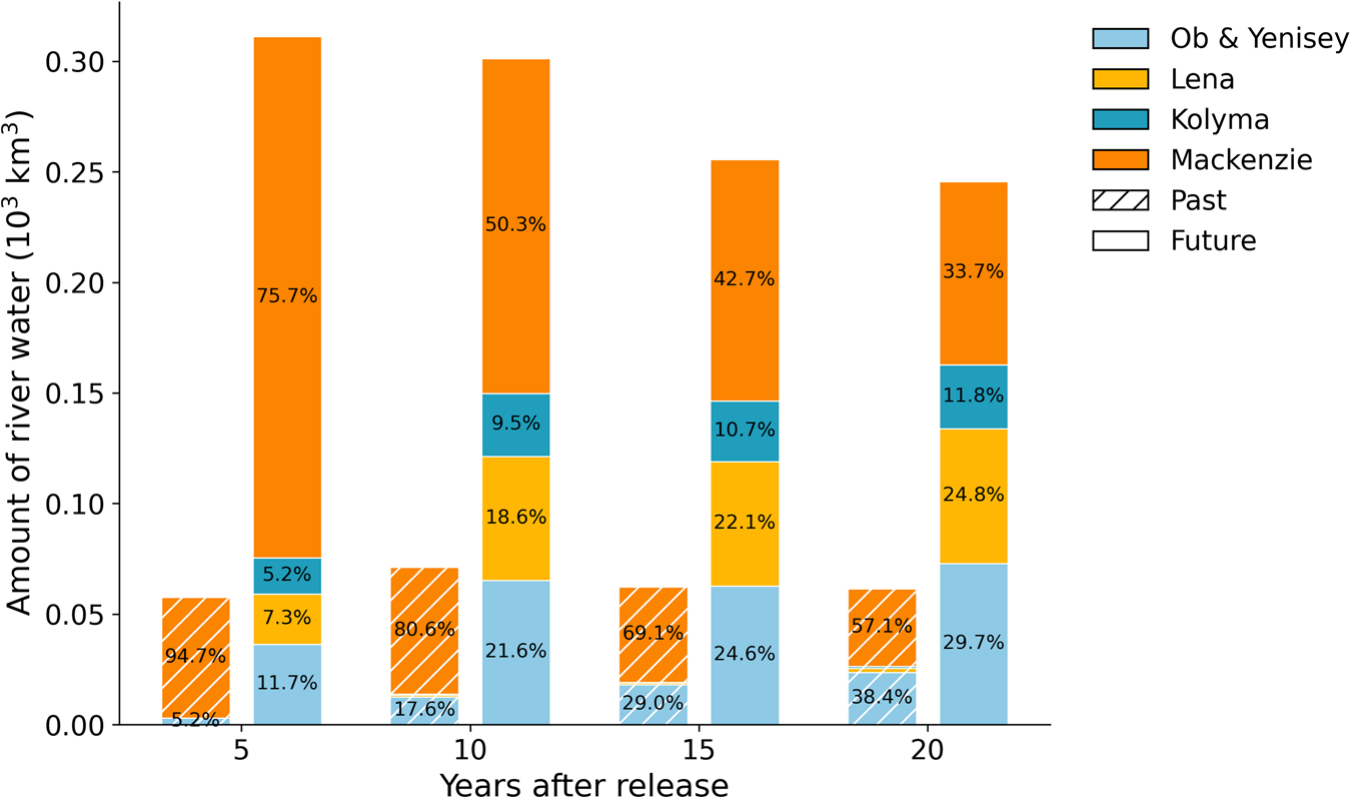
Amount and percentage of river water in the Canada Basin. The amount of river water refers to the runoff volume located in the Canada Basin originating from one year of river discharge. The percentage represents the proportion of river runoff contributed by each river relative to the total runoff from all the Arctic rivers considered.

The time series of river discharge volume and fraction in the Canada Basin quantify the future changes (Fig. 4a, b). During the historical period, only the Ob–Yenisey discharge from the Siberian shelf exhibits a noticeable fraction in the Canada Basin (Fig. 4a, b). Under future climate, river water from all major Siberian rivers shows increased penetration into the Canada Basin, with volumes reaching 30–70 km^3^ originating from one-year discharge (Fig. 4a), corresponding to 5–15% of their respective annual discharge (Fig. 4b). The volume of Mackenzie discharge in the Canada Basin decreases over time, yet remains larger than that of any individual Siberian river (Fig. 4a). However, the collective contribution of Siberian rivers to the total Arctic river water in the Canada Basin increases markedly under climate warming (Fig. 5). Its proportion rises from about 25% after five years to 50% after ten years, and continues to grow thereafter. As a result, the future Canada Basin will feature a more balanced mixture of North American and Eurasian inputs, both contributing more substantially than in the historical period.

Surface particle tracking experiments consistently show an increased accumulation of Siberian discharge in the Canada Basin under future climate (Fig. 4c–f). A larger fraction of surface particles released over the Siberian shelves penetrates into the Canada Basin, reaching 20–45%, compared with the 5–15% fractions indicated by the online tracers. This suggests that Siberian riverine materials floating near the surface have higher chances to be accumulated into the Canada Basin than those sinking to greater depths, likely because surface Ekman transport is largely restricted to the upper 10–20 m^52^. Furthermore, the spreads among the ensemble of particle tracking experiments reveal substantial interannual variability in particle fate (Fig. 4c–f), indicating that the convergence of Siberian riverine water and floating materials into the western Arctic occurs not with a steady efficiency. Due to surface Ekman convergence in the Beaufort Gyre region, river waters and drifting particles that enter the region tend to remain within the gyre for long periods (Figs. 4, 5). As a result, the Canada Basin will become a hub of pan-Arctic accumulation in a warming climate.

Particles representing river discharge from the Barents Sea also show increased accumulation in the Canada Basin under climate warming (Supplementary Fig. 4), although the corresponding fractions are lower than those of the four major river systems discussed above (Supplementary Fig. 5).

To assess the robustness of our findings, we also conducted particle tracking experiments for the CMIP6 SSP245 scenario. Under both the SSP245 and SSP585 scenarios, the particles released from the Siberian rivers show clearly increased accumulation in the Canada Basin relative to the historical period, with the warmer SSP585 scenario leading to even greater accumulation of Siberian particles (Supplementary Fig. 5).

### Increasing connectivity associated with multi-scale circulation changes

The mean ocean current speed in the upper 100 m of the Arctic Ocean is projected to intensify by 73% during the 21st century under the SSP585 scenario, with the strongest increase occurring in the Beaufort Gyre, the Transpolar Drift, and the shelfbreak current (Fig. 2a, b). Arctic sea ice is projected to decline substantially, while near-surface winds are expected to strengthen (Supplementary Fig. 6). Enhanced air-sea momentum transfer driven by sea ice decline, together with intensified winds, promotes freshwater accumulation in the Canada Basin^47,48^, strengthening the Beaufort Gyre circulation (Fig. 2a, b and Supplementary Fig. 7).

Future warming and freshening over the Siberian shelves is expected to reduce shelf-water density^51^. This reduction shifts the ocean current toward shallower depths, as indicated by the velocity of the outflow from the shelves into the Eurasian Basin (Supplementary Fig. 8). As a result, the shelfbreak current along the upper continental slope intensifies and feeds an accelerated Transpolar Drift (Fig. 2a, b). As the upper branch of the Arctic Circumpolar Boundary Current, the shelfbreak current plays an important role in transporting upper-ocean water masses^53^. We find that, under future warming, the accelerated shelfbreak current and Transpolar Drift enhance upper-ocean connectivity between the Siberian shelves and the subarctic seas, reducing the timescale for Siberian river discharge to reach the central Arctic and the North Atlantic Ocean (Figs. 1 and 3).

In addition to the intensification of the mean flow, mesoscale eddy activity in the upper Arctic Ocean is projected to increase (Fig. 2c, d), owing to reduced surface dissipation associated with sea ice decline and enhanced potential energy from increasing freshwater content^46^. In our simulations, Arctic eddy kinetic energy in the upper 100 m increases by about threefold under the considered warming condition (Supplementary Fig. 9). Eddies play an important role in mediating water-mass exchange across the Transpolar Drift, thereby linking the Eurasian and Canada basins^54^. As illustrated by the Lena River discharge, riverine water remains largely confined within the relatively narrow Transpolar Drift when eddy activity is weak in the historical period, whereas its distribution broadens substantially under future warming (Fig. 2e, f). An increase in eddy activity means intensified lateral stirring^46,55^. The intensified eddy stirring, evident in the simulated tracer field, leads to more efficient cross-front dispersion of riverine water (Fig. 2d, f).

On the one hand, intensified eddy activity promotes the dispersion of Siberian river discharge from the Transpolar Drift into the Amerasian Basin, where it becomes available for accumulation into the Beaufort Gyre. On the other hand, ocean surface stress is projected to increase due to continued sea ice decline and strengthening winds^47,48,56^ (Supplementary Fig. 6), enhancing Ekman convergence and thus the accumulation of surface waters in the Beaufort Gyre. Together, these processes — intensified eddy activity across the Transpolar Drift and enhanced Ekman convergence in the western Arctic — facilitate the accumulation of Siberian river discharge in the Beaufort Gyre, as described in the previous section. The presence of Siberian river particles in the Canada Basin increases from the SSP245 scenario to the warmer SSP585 scenario, whereas the accumulation of Mackenzie River particles is similar between the two scenarios (Supplementary Fig. 5). The further increase in the contribution of Siberian river particles to the Beaufort Gyre under stronger warming, in contrast to the saturated local contribution from the Mackenzie River, highlights the growing importance of enhanced eddy-induced dispersion across the Transpolar Drift.

## Discussion

Our results reveal a reorganization of Arctic river discharge under climate warming, owing to multi-scale changes in ocean circulation. The projected intensification of the shelfbreak current, associated with a reduction in shelf-water density, feeds into the Transpolar Drift and accelerates the export of Siberian river discharge toward the central Arctic and the North Atlantic. Simultaneously, the Canada Basin emerges as a new focal region for the accumulation of Siberian riverine water and drifting materials. The combined effects of enhanced eddy stirring across the Transpolar Drift and strengthened Ekman convergence over the Canada Basin transform the Beaufort Gyre from a largely regional reservoir of river discharge into a pan-Arctic convergence zone. This shift indicates increased cross-basin connectivity, enhancing the possibility that riverborne nutrients, carbon, and pollutants from Eurasia will accumulate extensively in the western Arctic, as demonstrated by our analysis of tracers and particles.

This study underscores the sensitivity of Arctic connectivity to multi-scale circulation changes. The interplay between large-scale mean flows, mesoscale eddies, and surface convergence governs the dispersal and accumulation of riverine inputs in a rapidly warming Arctic. Eddies are known to play crucial roles in transporting heat, salt and nutrients in the world ocean^57,58^. In the historical Arctic, extensive sea ice cover suppressed eddy activity^46,59,60^, limiting connectivity across the Transpolar Drift. In a future warming climate with reduced sea ice cover, the pronounced increase in eddy activity is expected to fundamentally reshape the spatial dispersal of water masses and drifting particles. In concert with large-scale circulation changes, the strengthening of eddy-driven exchange will substantially alter the landscape of drifting matter in the Arctic.

The circulation-driven redistribution of freshwater and terrigenous materials has far-reaching implications for the Arctic and subarctic seas. Enhanced lateral exchange between the Eurasian and Amerasian basins may blur biogeochemical boundaries, fostering new ecological linkages across the Arctic. Furthermore, increasing convergence of pan-Arctic pollutants, such as microplastics and industrial substances, into the western Arctic raises concerns for both marine ecosystems and local communities. The persistence of drifting contaminants within the gyre could lead to prolonged ecological exposure, as some of these materials could be retained for decades. Because some freshwater in the Beaufort Gyre is episodically released on quasi-decadal timescales^61^, the associated export of trapped contaminants to the North Atlantic during these release events could trigger abrupt ecological impacts downstream. Moreover, the accelerated export of Siberian discharge shortens the timescales over which terrigenous materials start to interact with ecosystems in the central Arctic and subarctic seas.

Our experiments represent two classes of drifting particles, those with seawater-like density that mix and advect similarly to seawater, and those that remain near the surface. They demonstrate different lateral dispersion depending on their vertical distribution. In reality, terrigenous materials span a wide range of densities and may undergo degradation, biogeochemical transformation, biofouling, or sedimentation^22,62,63^. Sea ice, when present, serves as an important carrier for some floating materials^64,65^, and surface waves can further influence the transport of large floating debris^66^. Consequently, the fate of riverine materials is complex, and predicting their future trajectories requires consideration of the distinct physical and biogeochemical properties of each material type. Furthermore, unknown future variations in the composition and load of drifting materials from different sources introduce substantial uncertainty in predicting their concentrations in the ocean. Nevertheless, the main findings of this study — enhanced ocean connectivity arising from multi-scale circulation changes and the emergence of a pan-Arctic accumulation hub in the western Arctic — remain robust. These changes are expected to control the distribution of drifting materials that remain entrained in ocean currents, also including those of non-terrigenous origin. Materials that tend to settle during transport are also likely to spread more widely across the seafloor due to accelerated and broadened dispersion before deposition.

The numerical simulations used in this study are eddy-permitting in the Arctic and may therefore underestimate the full impact of mesoscale eddies, highlighting the need for further investigations using higher resolutions. In addition, simulations of river runoff in climate models have large uncertainties^67^, which emphasizes the need for improved runoff projections to better constrain future changes and their climatic and ecological impacts.

## Methods

### Numerical model simulations

Historical (1900–2014) and future projection (2015–2100) simulations were performed using the Finite volumE Sea ice-Ocean Model (FESOM2)^68,69^. Both the ocean and sea ice components of FESOM2 are formulated on unstructured grids, allowing the model resolution to be refined in areas of interest. FESOM2 realistically represents the mean state and variability of the Arctic Ocean hydrography and circulation compared to observations and other models^70^. In this study, the horizontal resolution in the Arctic region is 4.5 km and is coarsened elsewhere to reduce computational cost (Supplementary Fig. 10a). The largest eddies, arising from baroclinic instability, are expected to be resolved in the Arctic deep basin, but not over the shelves and along the continental slopes (Supplementary Fig. 10b, c). The chosen resolution represents a compromise between our requirements and computational constraints. The vertical grid spacing is 5 m near the surface and gradually increases downwards, with 47 z-levels in total.

The atmospheric forcing and river runoff used to drive FESOM2 were derived from climate simulations with the Earth system model FIO-ESM v2.1 under the CMIP6 SSP585 scenario^71^. To reduce the impacts of systematic biases in the atmospheric forcing on the FESOM2 simulations, climatological 3-hourly mean biases relative to atmospheric reanalysis data were subtracted from the forcing fields, including near-surface winds and air temperature^72^. The FESOM2 simulations reasonably reproduce the ocean and sea ice conditions in the Arctic, with substantially improved performance compared with CMIP6 models^72^. In particular, the Beaufort Gyre, which often unrealistically extends across the entire Arctic basin in CMIP6 models, is well captured in the FESOM2 simulations, as indicated by the spatial pattern of freshwater content (Supplementary Fig. 11). This improvement is critical for the present study, which requires decent simulations of Arctic Ocean circulations, including the positions of major currents.

To investigate the circulation of Arctic river discharge, we introduced passive tracers to represent river runoff in our model simulations. We focused on the four largest Arctic river systems: the Ob–Yenisey (Kara Sea), Lena (Laptev Sea), Kolyma (East Siberian Sea), and Mackenzie (Beaufort Sea), assigning one passive tracer to each. For each shelf sea, all river discharge was included in the tracer associated with the representative major river. As discharge from these major rivers dominates over that from other rivers, each tracer is referred to by the corresponding major river, although it represents the total runoff from the entire shelf sea.

We took two simulation periods: a historical period (1985–2004) and a future period (2071–2090), corresponding to the periods of the first ensemble member of the Lagrangian particle tracking experiments described below. The tracers were initialized with a zero value and received a surface flux during the first year, equal to the respective river runoff. They were subject to the same mixing and advection processes as salinity and temperature, allowing them to represent the dispersion of riverine waters throughout the simulations. The vertically integrated tracer values (in meters) represent the column content of the river runoff at each location, while their volume integration (in cubic kilometers) quantifies the total volume of river water in a considered region.

### Particle tracking experiments

The passive tracers in the numerical simulations represent riverine waters and drifting materials that follow the dynamical vertical distribution of riverine inflows. To complement these simulations, we conducted Lagrangian surface particle tracking experiments using the Parcels software^73^. In this case, the particles represent floating materials that remain near the surface. Comparing the two approaches allows us to assess how the vertical distribution of materials influences their lateral dispersion.

The particle tracking experiments were driven by surface velocity fields derived from the FESOM2 simulations described above with an hourly time step. Particles were released at the river mouths of the four major rivers, as indicated by the red bars in Supplementary Fig. 1. Releases occurred during the first experiment year, and the particles were tracked for 20 years. The number of particles released in each month is scaled according to the seasonal variability of river runoff. After the particles entered the Nordic Seas, they were prevented from reentry into the Arctic Ocean.

During the historical period, 1000 particles were released for each river, while 1350 particles were released in the future scenario to account for the projected  ~ 35% increase in Arctic river runoff in CMIP6 multi-model mean^74^ and in the runoff forcing derived from FIO-ESM v2.1. Because the concentrations of drifting materials in river runoff are unknown and vary with material type, river, and future changes, particle distributions in the ocean were analyzed in terms of fractions, that is, the fraction of particles entering the Canada Basin relative to the total number released from each river. In this approach, the absolute number of released particles does not affect the results as long as the sample size is sufficiently large. We performed sensitivity experiments using 1000 particles in the future scenario. The resulting spatial distribution and the fraction of particles in the Canada Basin are comparable to those obtained with 1350 particles (Supplementary Figs. 2 and 12). The differences between these experiments (Supplementary Figs. 2 and 12) are much smaller than the climate change signal (Fig. 4c–f and Supplementary Fig. 1).

We performed 11 particle tracking experiments for each river and each period, initialized in different starting years. For the historical period, the starting years range from 1985 to 1995, and for the future scenario, from 2071 to 2081. As each experiment lasted for 20 years, the 11 experiments in the historical period span 1985–2014, which represents the present-day condition. The 11 experiments in the future scenario span 2071–2100, which represents the long-term-future condition. The two ensembles of particle tracking experiments for each river allow us to assess the interannual variation of the dispersion of river discharge.

The four major river systems considered above represent the four primary Arctic shelf seas but do not include the Barents Sea. Although discharge from the two largest rivers in the Barents Sea sector (the Pechora and the Northern Dvina via the White Sea) is substantially smaller than that of the four major Arctic rivers, we conducted particle tracking experiments for these rivers to assess the future evolution of Barents Sea discharge pathways. With these additional experiments, we can get an overview of all shelf seas inside the Arctic domain (enclosed by the Fram Strait, Barents Sea Opening, Bering Strait and the northern boundary of the Canadian Arctic Archipelago). By comparing the fraction of particles entering the Canada Basin relative to the total number released, we can reveal how drifting matter from different shelf seas is differently influenced by climate change.

A future projection simulation with FESOM2 was also conducted under the CMIP6 SSP245 warming scenario. In contrast to SSP585, which is the upper-bound emission scenario often described as “fossil-fueled development” with no climate mitigation, the SSP245 scenario represents a “middle of the road” future with moderate climate protection measures. This simulation is identical to the SSP585 simulation described above, except for the scenario forcing. Although river runoff tracers are not available in this simulation, particle tracking experiments were conducted in the same manner as for the SSP585 scenario. A comparison between the two scenarios shows that stronger warming leads to a greater accumulation of Siberian river particles in the Canada Basin (Supplementary Fig. 5). This study focuses on the SSP585 scenario, and all results refer to SSP585 unless SSP245 is explicitly mentioned.

### Data analysis

We analyzed the distribution of tracers and particles in different regions, including the Arctic shelves, Arctic deep basin, and Canada Basin, as indicated in the inset in Fig. 3. The shelf and deep basin regions were separated by the 500 m isobath. The Arctic Ocean, including both the shelf and deep basin areas, was defined by the northern boundary of the Canadian Arctic Archipelago and the gateways of the Bering Strait, Fram Strait and Barents Sea Opening. The Canada Basin was bounded by the 500 m isobath to the south and east, by 180^∘^W to the west, and by 83^∘^N to the north.

We calculated the eddy kinetic energy (EKE) and freshwater content (FWC) in the numerical simulations. The ocean velocity is decomposed to mean and eddy components, $(u,v)=(\overline{u}+{u}^{{\prime} },\overline{v}+{v}^{{\prime} })$. The EKE is defined as

1 $$\,{{{\rm{EKE}}}}\,=\frac{1}{2}\left(\overline{{u}^{{\prime} 2}}+\overline{{v}^{{\prime} 2}}\right)=\frac{1}{2}\left(\overline{{u}^{2}}+\overline{{v}^{2}}\right)-\frac{1}{2}\left({\overline{u}}^{2}+{\overline{v}}^{2}\right),$$

where $\overline{{u}^{2}}$, $\overline{{v}^{2}}$, $\overline{u}$ and $\overline{v}$ are monthly-mean quantities saved from the model directly.

The FWC is defined as

2 $$\,{{{\rm{FWC}}}}=\int _{D}^{{{{\rm{surface}}}}}({S}_{{{{\rm{ref}}}}}-S)/{S}_{{{{\rm{ref}}}}}{{{\rm{d}}}}\,z,$$

where *S* is salinity, *S*_ref_ is the reference salinity (here, the Arctic mean salinity of 34.8^3^), and *D* is the depth of the isohaline corresponding to *S*_ref_. Because density variations in the Arctic basin are largely determined by salinity variations, the spatial pattern of FWC also reflects the structure and position of the major circulation features (Supplementary Fig. 7).

### Model evaluations

To assess the model simulations used in this study, we employ gridded hydrography data from the PHC3 observations^75^, sea ice satellite observations^76^, model output from the Coupled Model Intercomparison Project phase 6 (CMIP6), and high-resolution simulations from the Ocean Model Intercomparison Project phase 2 (OMIP2)^77,78^. The CMIP6 simulations include the first realizations of the following models: ACCESS-CM2, AWI-CM-1-1-MR, CESM2-WACCM, EC-Earth3, FIO-ESM-2-0, INM-CM5-0, MPI-ESM1-2-LR, MPI-ESM1-2-HR, NorESM2-LM, NorESM2-MM, and UKESM1-0-LL. The high-resolution OMIP2 models considered here are ACCESS-MOM, CMCC-NEMO, FSU-HYCOM and IAP-LICOM, which were described in previous Arctic Ocean model intercomparison studies^70^. These OMIP2 models employ spatial resolutions comparable to that used in our FESOM2 experiments, but they are driven by atmospheric reanalysis data^77^) rather than by forcing derived from climate models.

Arctic large-scale circulations, particularly the Transpolar Drift and the Beaufort Gyre, are the primary pathways for the distribution and export of Arctic river runoff. It is therefore essential that numerical models adequately represent these circulation systems. The spatial pattern of freshwater content is a useful diagnostic of the Arctic large-scale circulation^48^, as sea surface height and density variations in the Arctic basin are mainly determined by salinity changes. The PHC3 climatology shows a well-defined Beaufort Gyre with elevated freshwater content located in the western Arctic, as well as a pronounced freshwater gradient between the Eurasian and Amerasian basins associated with the Transpolar Drift (Supplementary Fig. 11a). Our high-resolution FESOM2 simulations reproduce both the spatial distribution and magnitude of the observed freshwater content reasonably well (Supplementary Fig. 11b). In contrast, the CMIP6 models simulate an overly extensive Beaufort Gyre that spans nearly the entire Arctic basin (Supplementary Fig. 11c). The high-resolution OMIP2 simulations also struggle to capture the observed strength and geographical position of the Beaufort Gyre and the Transpolar Drift (Supplementary Fig. 11d–g), despite being forced with realistic atmospheric reanalysis fields, unlike the climate model-simulated atmospheric fields used in our FESOM2 simulations.

Averaged over the Eurasian and Amerasian basins, the salinity profiles indicate that ocean models tend to simulate a weaker halocline compared to the observations (Supplementary Fig. 13). CMIP6 models exhibit a wide spread in their simulated salinity fields^79^, and even the multi-model mean retains substantial biases in the salinity profiles across the models in our comparison (Supplementary Fig. 13). In contrast, the FESOM2 simulations provide a reasonable representation of the salinity profiles. Notably, when FESOM2 is forced with atmospheric reanalysis data as in the high-resolution OMIP2 framework, the model biases are further reduced relative to those in the simulations used here^70^. This suggests that part of the FESOM2 biases arises from the atmospheric forcing, which is derived from coupled climate model outputs rather than from observationally constrained reanalysis fields (see model description at the beginning of the Method section).

Consistent with the reasonable representation of large-scale Arctic Ocean circulations in our simulations (Supplementary Fig. 11), the Siberian river discharge is transported across the central Arctic along the Transpolar Drift, as demonstrated by the passive tracers (Fig. 1). Because Siberian river discharge supplies freshwater to the central Arctic, the upper ocean salinity in this region also provides a useful indicator of the river discharge pathway. Vertical transects across the central Arctic show that FESOM2 simulations largely reproduce the observed salinity structure (Supplementary Fig. 14a, b). In contrast, the high-resolution OMIP2 simulations tend to exhibit clear salinity biases in the central Arctic: some models show low salinity, implying that the Transpolar Drift, and thus the runoff pathway, is displaced toward the Eurasian continental shelf (Supplementary Fig. 14c, f and Supplementary Fig. 11d, g), whereas some display positive salinity biases, suggesting a shift of the Transpolar Drift towards the Amerasian Basin (Supplementary Figs. 14d, 11e). Most of the riverine water contributing to the surface freshwater content remains within the upper hundred meters^1–3^, a vertical structure that is also well reproduced in our simulations (Supplementary Fig. 15). Although the passive tracers used here do not allow us to quantify the stabilized fraction of river water because they were released only during the first year of tracer experiments for our purpose, their vertical and lateral distribution patterns are consistent with recent in-situ observations employing chemical tracers^35^.

The river runoff used in our simulations is derived from the FIO-ESM v2.1 output^71^. For consistency, we use runoff fields from this model for both the historical and future scenarios. During the historical period, the FIO-ESM v2.1 runoff is comparable to the observation-based climatological estimate^80^, while being lower than another estimate combining observations and model simulations^81^ (Supplementary Fig. 16). It is also very close to the CMIP6 multi-model mean in terms of both magnitude and future evolution. We note that available reference estimates of Arctic river runoff are subject to substantial uncertainty due to sparse gauging networks and the need to fill ungauged regions and periods using extrapolation and model simulations^80,81^.

In the historical period, FESOM2 adequately reproduces both the satellite-observed magnitudes and declining trends in Arctic summer and winter sea ice extent (Supplementary Fig. 17). The coarse-resolution CMIP6 models show large inter-model spreads in both magnitudes and trends.

In summary, although it is widely recognized that state-of-the-art numerical models face considerable challenges in adequately simulating the hydrography and circulation of the Arctic Ocean^48,79,82^, our high-resolution FESOM2 simulations demonstrate notable improvements. These improvements enable a more reliable investigation of future changes in Arctic Ocean circulations and their consequences for the dispersal pathways of river discharge and drifting matter.

## Supplementary information

Supplementary Information

Transparent Peer Review file

## Data Availability

The model data used to produce the paper figures are available at 10.5281/zenodo.19666191.

## Code availability

The code for generating paper figures is available at 10.5281/zenodo.19666191.
