# Supplementary material for: Warming transforms the western Arctic Ocean into a hub of drifting matter
Source: Nat Commun. 2026 Jun 16;17:5317. doi: 10.1038/s41467-026-74439-5 (PMC13272592; doi:10.1038/s41467-026-74439-5)
Supplement: Supplementary file 1 — Supplementary Information [file 41467_2026_74439_MOESM1_ESM.pdf]

Supplementary Information for
Warming transforms the western Arctic Ocean into a hub of drifting matter

Kou Wang[1,2†], Caili Liu[3,4†], Qi Shu[3,4†], Claudia Wekerle[2], Caixia Wang[1], Qiang Wang[2*]

[1]College of Oceanic and Atmospheric Sciences, Ocean University of China, Qingdao, 266100, China.
[2*]Alfred Wegener Institute, Helmholtz Centre for Polar and Marine Research, Bremerhaven, 27570, Germany.
[3]First Institute of Oceanography and Key Laboratory of Marine Science and Numerical Modeling, Ministry of Natural Resources, Qingdao, 266061, China.
[4]Shandong Key Laboratory of Marine Science and Numerical Modeling, Qingdao, 266061, China.

*Corresponding author(s). E-mail(s): qiang.wang@awi.de;
†These authors contributed equally to this work.

Content

Supplementary Figures 1-17

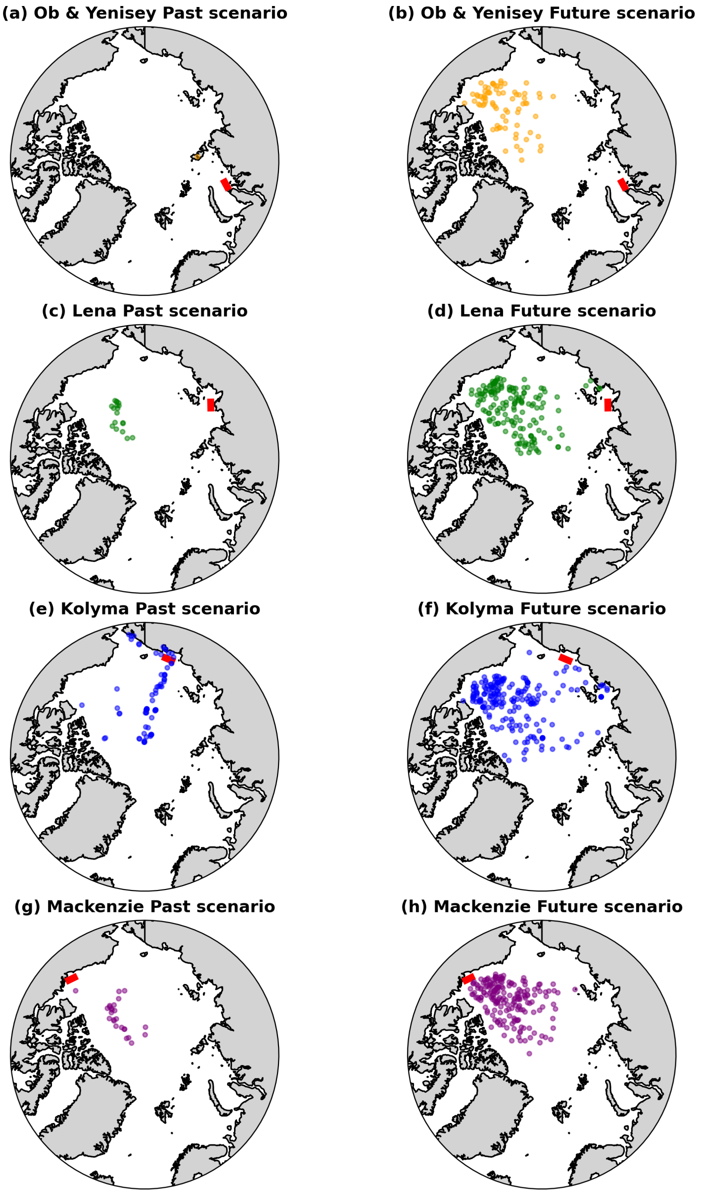

Supplementary Fig. 1: Spatial distribution of surface particles after being released for 20 years for the four major river systems. a,b Particles released from Ob–Yenisey rivers in the (**a**) historical and (**b**) future periods. **c,d** The same as (**a,b**), but for the Lena River. **e,f** The same as (**a,b**), but for the Kolyma River. **g,h** The same as (**a,b**), but for the Mackenzie River. The results from the first experiment ensemble member are shown, in which the particles are released in 1985 (**a,c,e,g**) and 2071 (**b,d,f,h**). Only every fourth particles are shown for a better illustration. The locations of particle release are indicated by red bars. The absence of particles in (**a**) indicates that all particles have exited the Arctic.

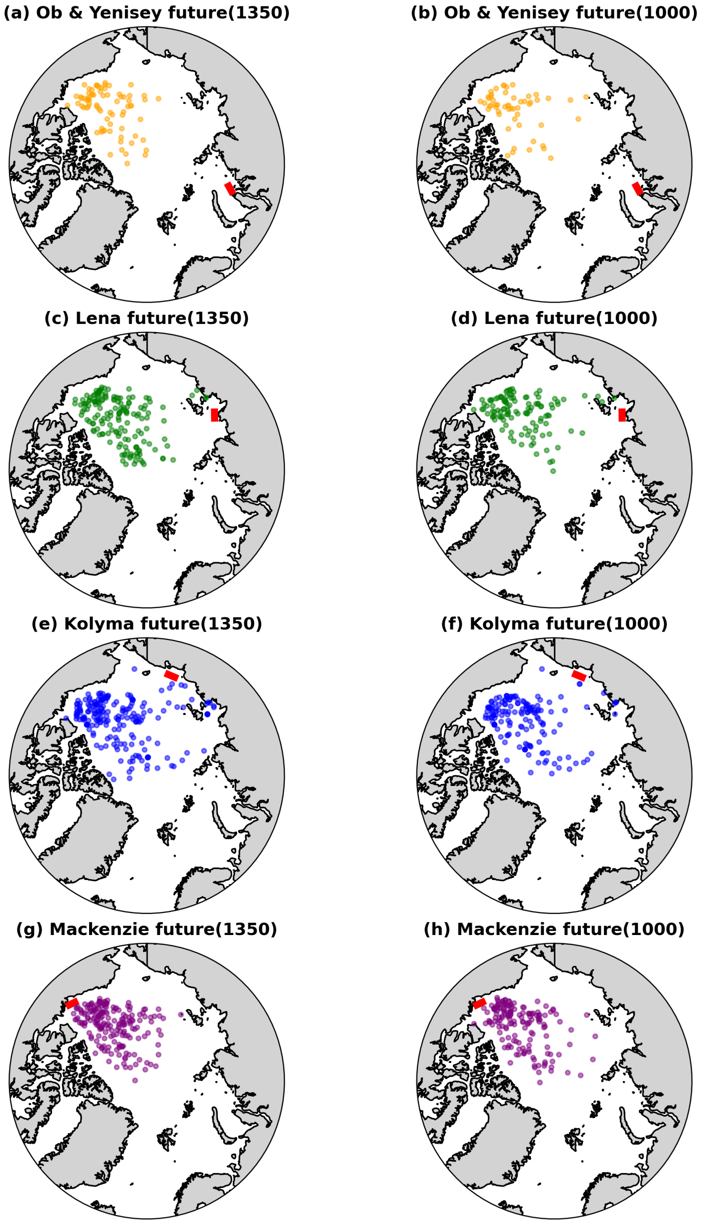

Supplementary Fig. 2: Spatial distribution of surface particles in experiments with different number of released particles. a,b Particles after being released for 20 years from Ob–Yenisey rivers in the experiment with (**a**) 1350 particles and (**b**) 1000 particles. The particles are released in 2071. **c,d** The same as (**a,b**), but for the Lena River. **e,f** The same as (**a,b**), but for the Kolyma River. **g,h** The same as (**a,b**), but for the Mackenzie River. Only every fourth particles are shown for a better illustration. The locations of particle release are indicated by red bars.

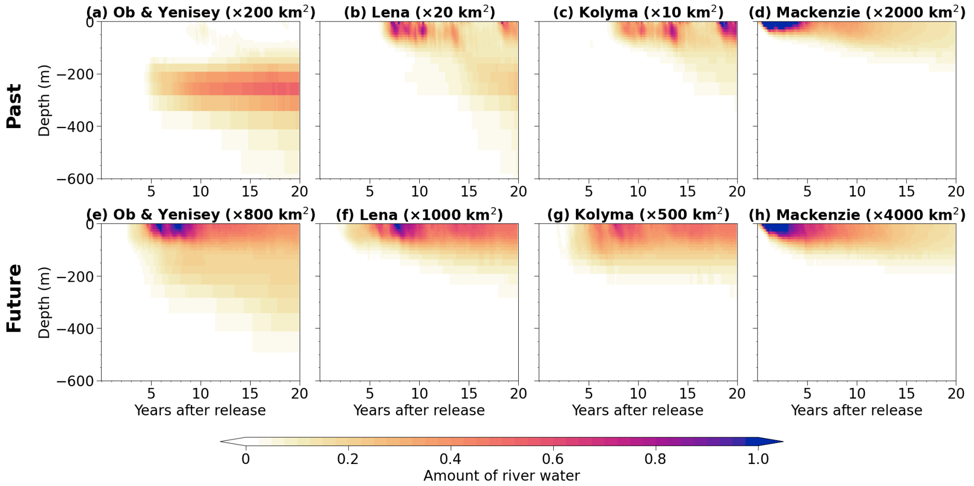

Supplementary Fig. 3: Depth-time plots of riverine water horizontally integrated over the Canada Basin. a-d Historical period. **e-h** Future period. Note that different scaling factors are applied as shown in panel titles in order to better illustrate the vertical structure for each case. The magnitudes of riverine water content in the Canada Basin are much larger in the future period than in the historical period for all the rivers.

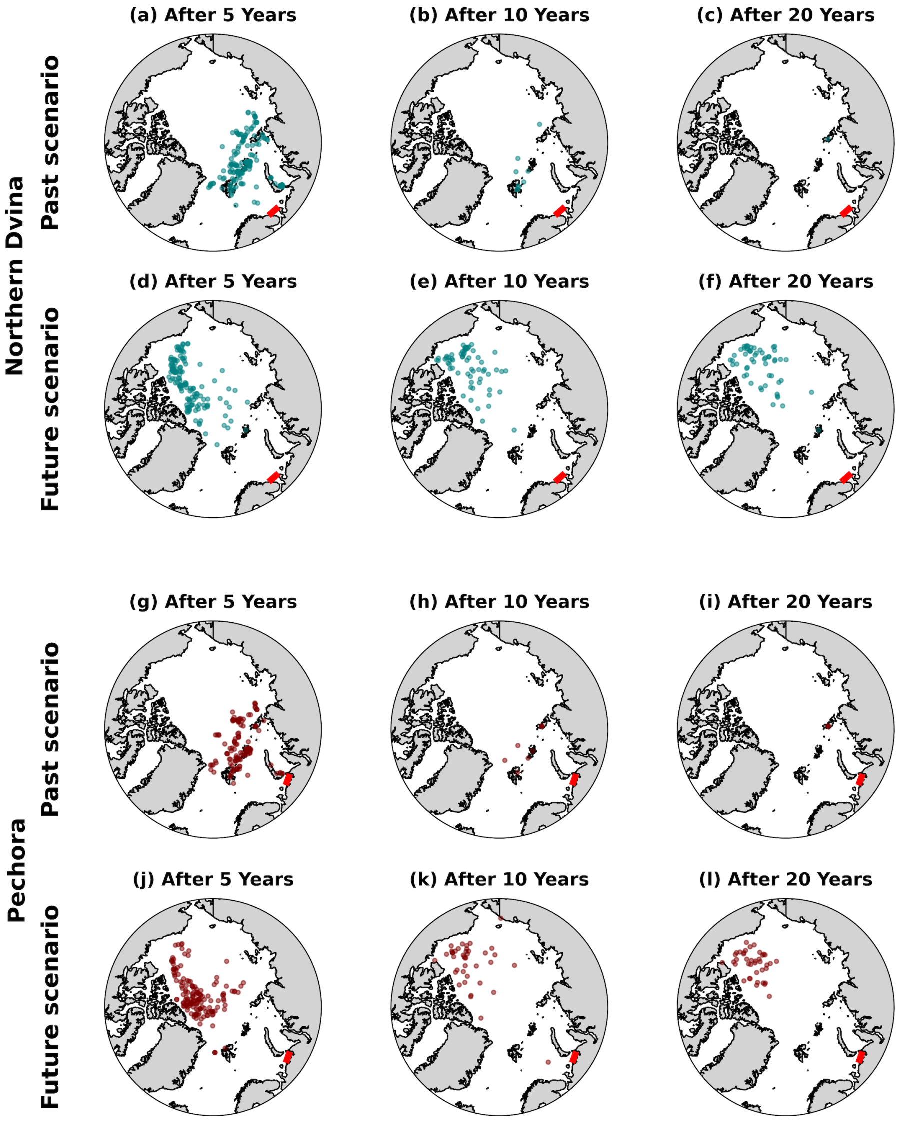

Supplementary Fig. 4: Spatial distribution of surface particles for the Barents Sea rivers. a-c Particles released from Northern Dvina in the historical period: after (**a**) 5 years, (**b**) 10 years, and (**c**) 20 years. **d-f** The same as (**a-c**), but for the future period. **g-l** The same as (**a-f**), but for the Pechora River. Only every fourth particles are shown for a better illustration. The locations of particle release are indicated by red bars. Northern Dvina particles are released at the White Sea exit. An absence of particles indicates that all particles have exited the Arctic at that time.

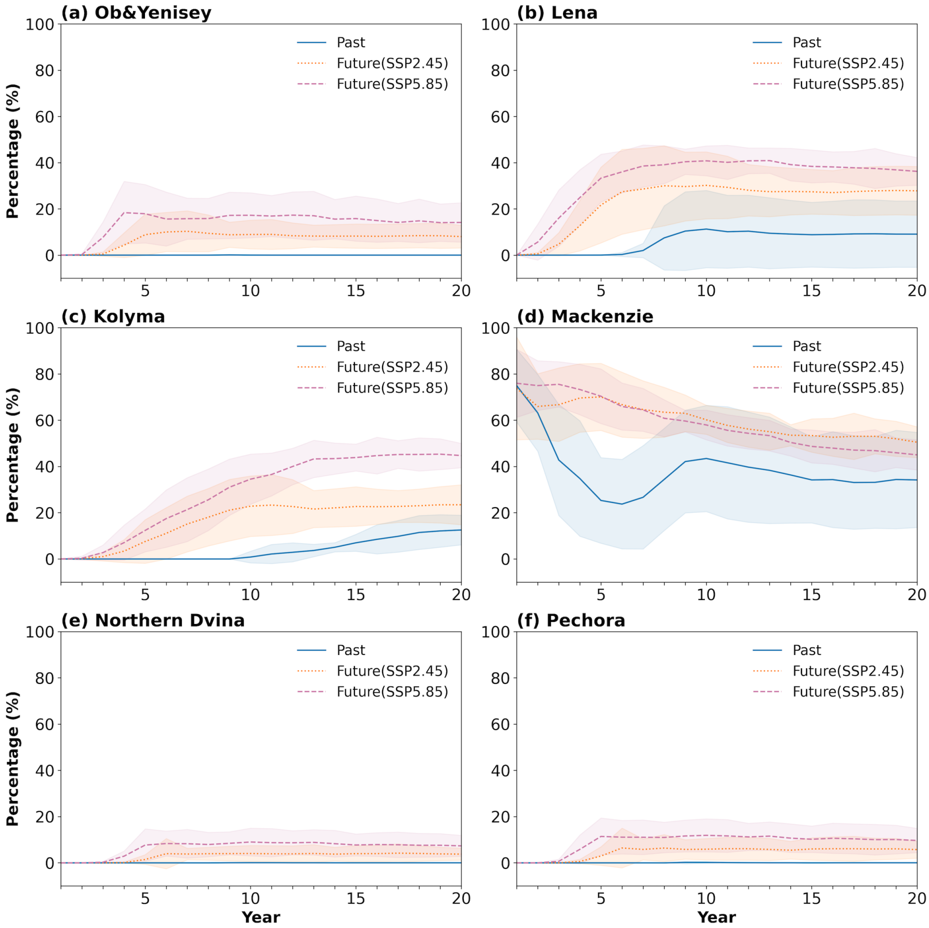

Supplementary Fig. 5: Proportion of surface particles in the Canada Basin relative to the total released particles in different CMIP6 scenarios. a-f Fraction of surface particles in the Canada Basin released from (**a**) Ob–Yenisey, (**b**) Lena, (**c**) Kolyma, (**d**) Mackenzie, (**e**) Northern Dvina, and (**f**) Pechora. The shading areas indicate one standard deviation of 11 particle tracking experiments.

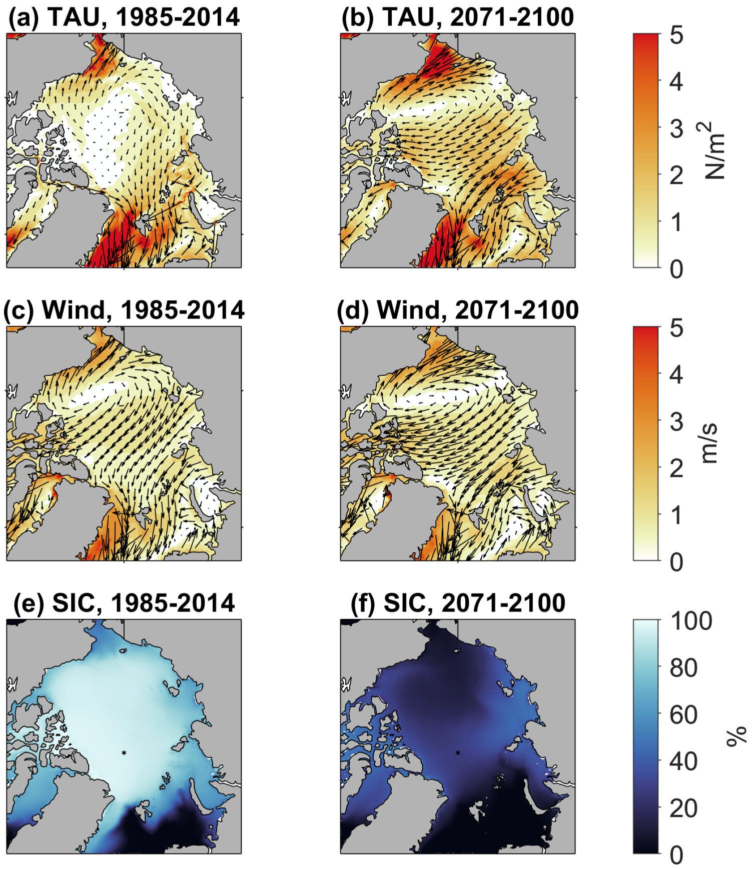

Supplementary Fig. 6: Increasing ocean surface stress associated with sea ice decline and wind strengthening. a,b Ocean surface stress (TAU) averaged over (**a**) 1985–2014 and (**b**) 2071–2100. **c,d** The same as (**a,b**), but for near-surface winds. **e,f** The same as (**a,b**), but for sea ice concentration (SIC).

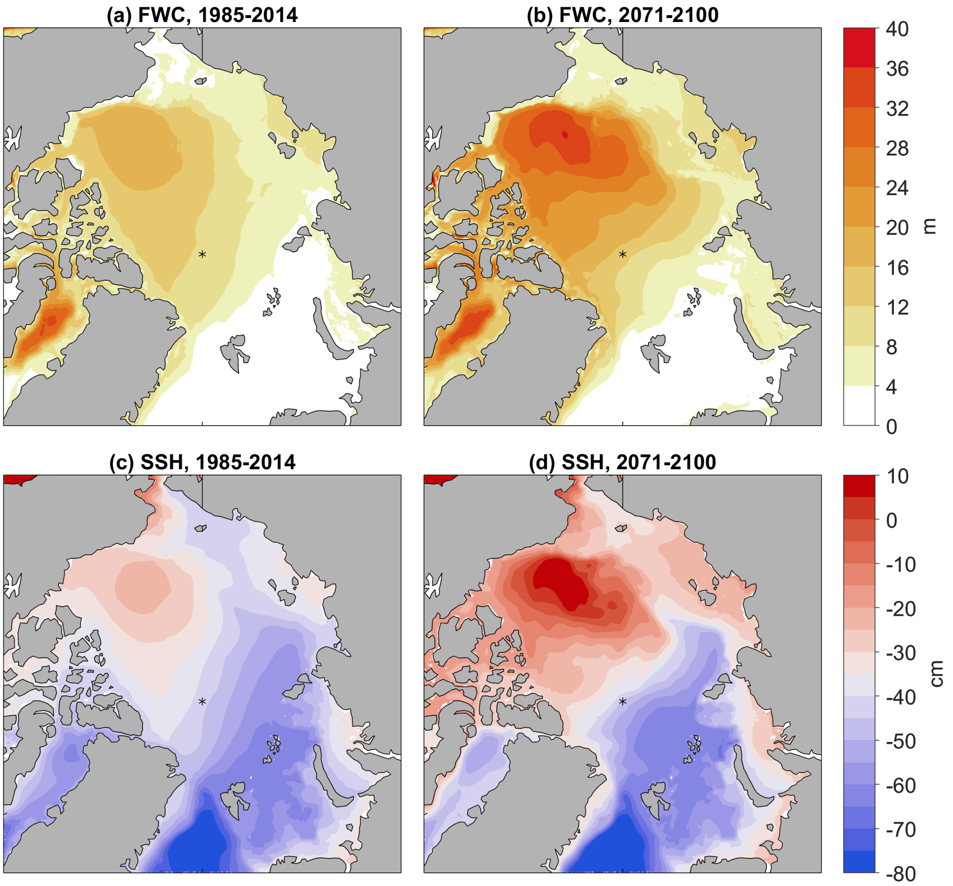

Supplementary Fig. 7: Freshwater content (FWC) and sea surface height (SSH) in different periods. a,b FWC averaged over (**a**) 1985–2014 and (**b**) 2071–2100. **c,d** The same as (**a,b**), but for SSH.

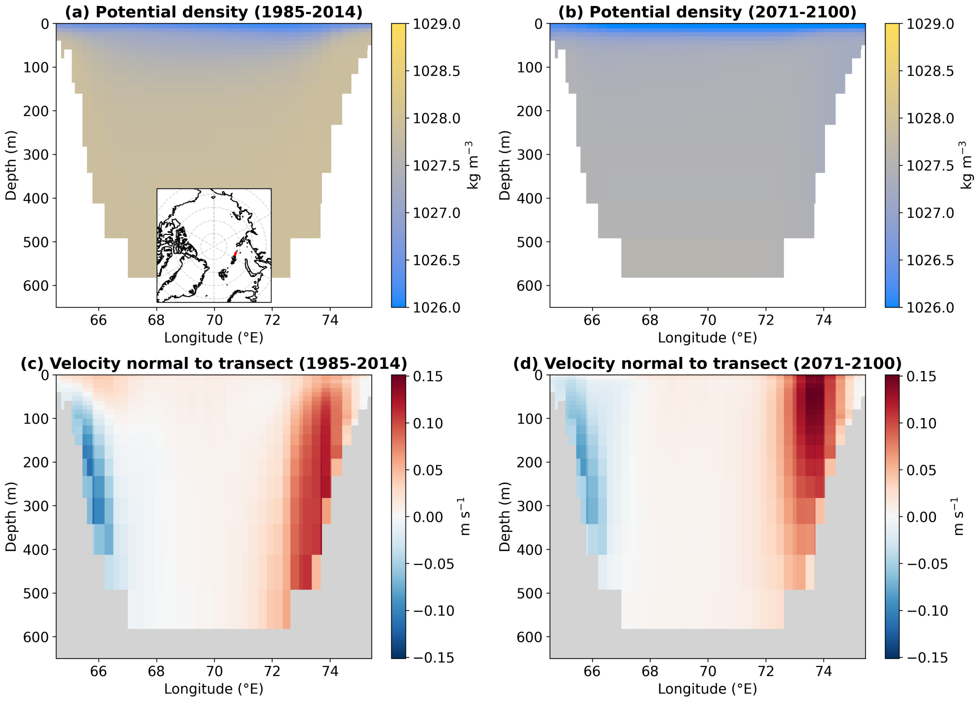

Supplementary Fig. 8: Decrease in density and associated upward shift of shelf water outflow in a warming climate. a,b Potential density across the St. Anna Trough in the (**a**) historical period and (**b**) future period. **c,d** The same as (**a,b**), but for velocity normal to the transect. Positive velocity indicates outflow from the shelf to the basin. The location of the transect is depicted in the inset in (**a**).

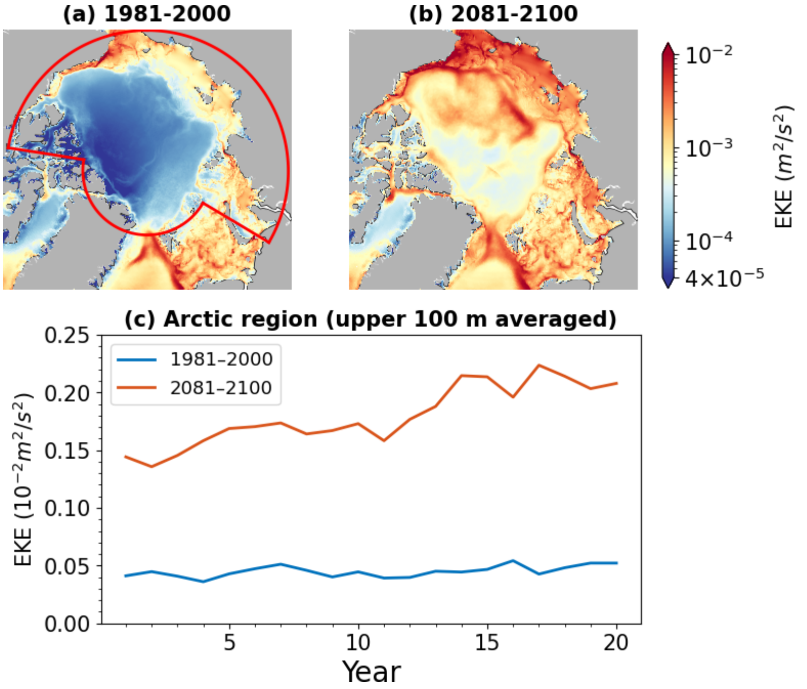

Supplementary Fig. 9: Increasing eddy activity in the upper Arctic Ocean in a warming climate. a,b Eddy kinetic energy (EKE) averaged over the upper 100 m in the period of (**a**) 1981–2000 and (**b**) 2081–2100. **c** Time series of Arctic Ocean mean EKE over the upper 100 m during the two periods. The red box in (**a**) shows the area used to calculate the Arctic Ocean mean EKE.

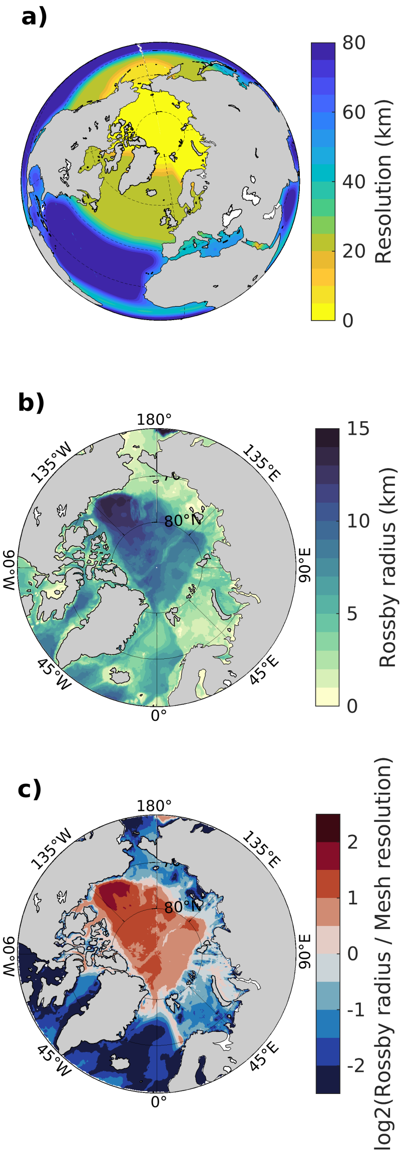

Supplementary Fig. 10: Model mesh resolution and first baroclinic Rossby radius. a Mesh resolution. **b** First baroclinic Rossby radius of deformation. **c** Ratio of first baroclinic Rossby radius to mesh resolution shown as $\log_2$ values. Eddy-permitting resolution requires the $\log_2$ value to be larger than zero.

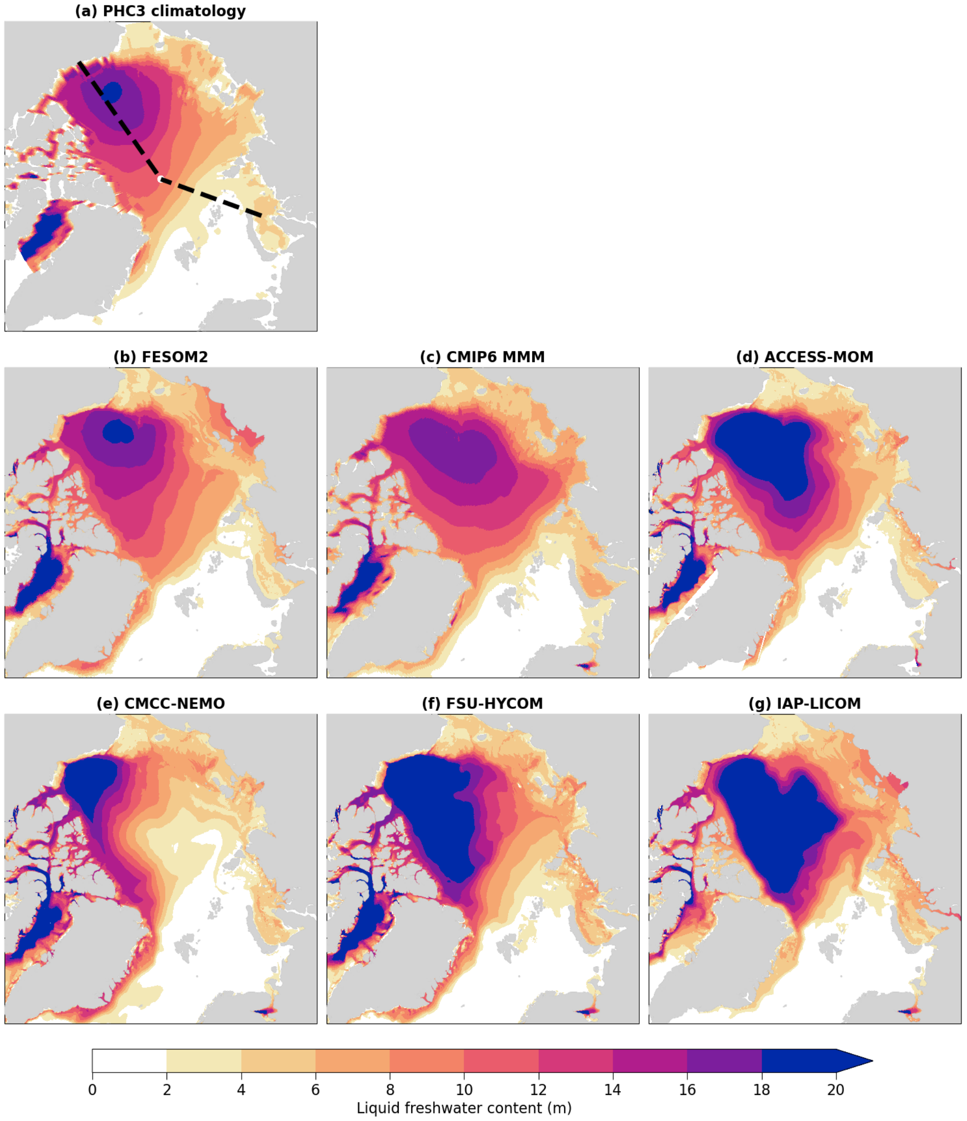

Supplementary Fig. 11: Improved representation of major Arctic Ocean features in high-resolution FESOM2 simulations. a-g Freshwater content (FWC) in (**a**) PHC3 climatology, (**b**) high-resolution FESOM2 simulations used in this study, (**c**) CMIP6 multi-model mean (MMM), and high-resolution OMIP2 simulations of (**d**) ACCESS-MOM, (**e**) CMCC-NEMO, (**f**) FSU-HYCOM, and (**g**) IAP-LICOM. Model results are the average over the period of 1981–2000. The black dashed lines in (**a**) denote the location of the vertical transect used in Supplementary Figs. 14 and 15.

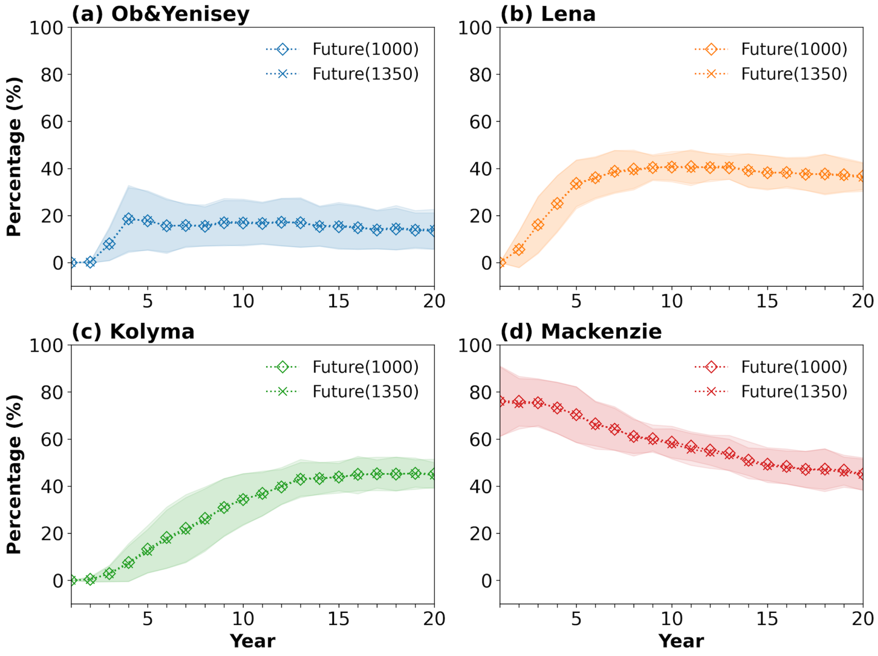

Supplementary Fig. 12: Proportion of surface particles in the Canada Basin relative to the total number of released particles for experiments with different numbers of released particles. a Ob–Yenisey. **b** Lena. **c** Kolyma. **d** Mackenzie. The shading areas indicate one standard deviation of 11 particle tracking experiments. In one set of experiments, 1350 particles were released from each river, while 1000 particle were released in the other set.

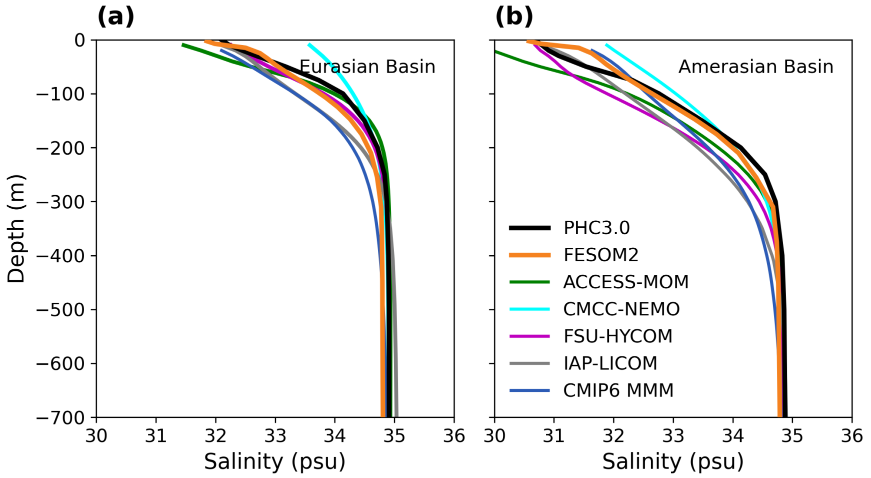

Supplementary Fig. 13: Evaluation of simulated salinity profiles. **a** Mean salinity profiles averaged in the Eurasian Basin. **b** Mean salinity profiles averaged in the Amerasian Basin. Model results are the average over the period of 1981–2000.

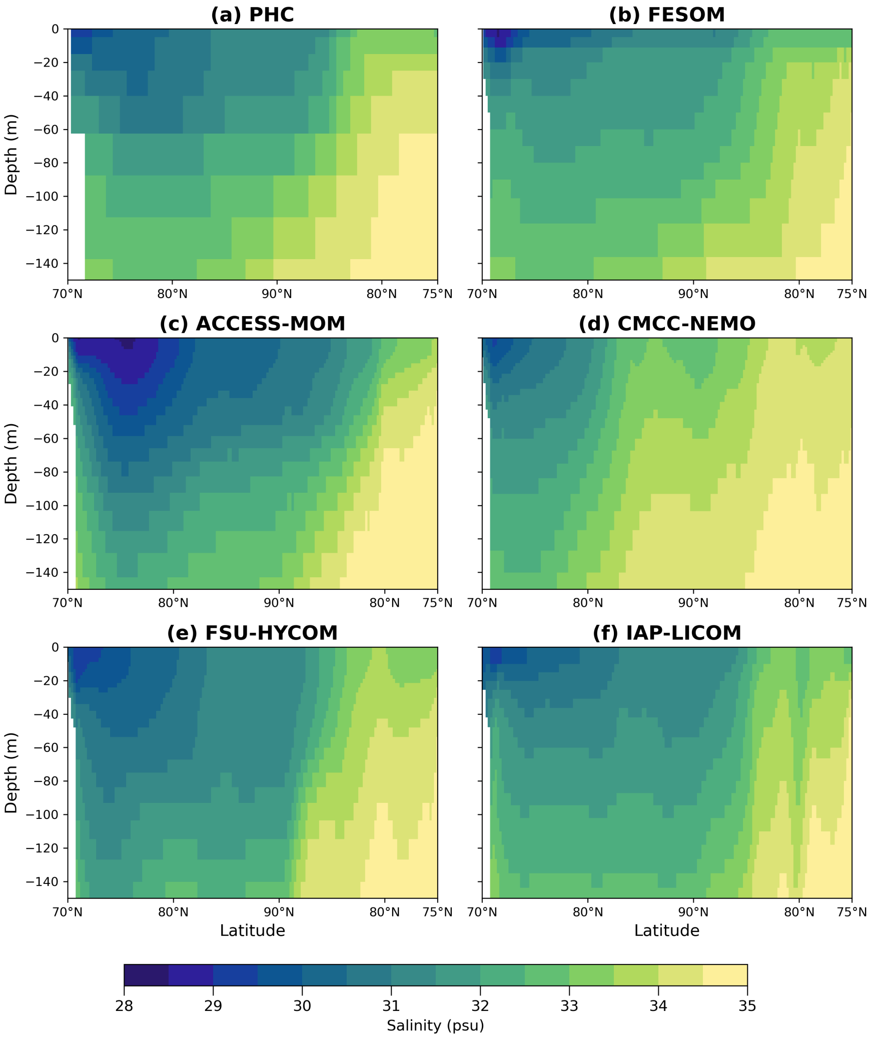

Supplementary Fig. 14: Salinity in a transect crossing the central Arctic. a-f Salinity in (**a**) PHC3 climatology, (**b**) high-resolution FESOM2 simulations used in this study, and high-resolution OMIP2 simulations of (**c**) ACCESS-MOM, (**d**) CMCC-NEMO, (**e**) FSU-HYCOM, and (**f**) IAP-LICOM. Model results are the average over the period of 1981–2000. The location of the transect is denoted in Supplementary Fig. 11a.

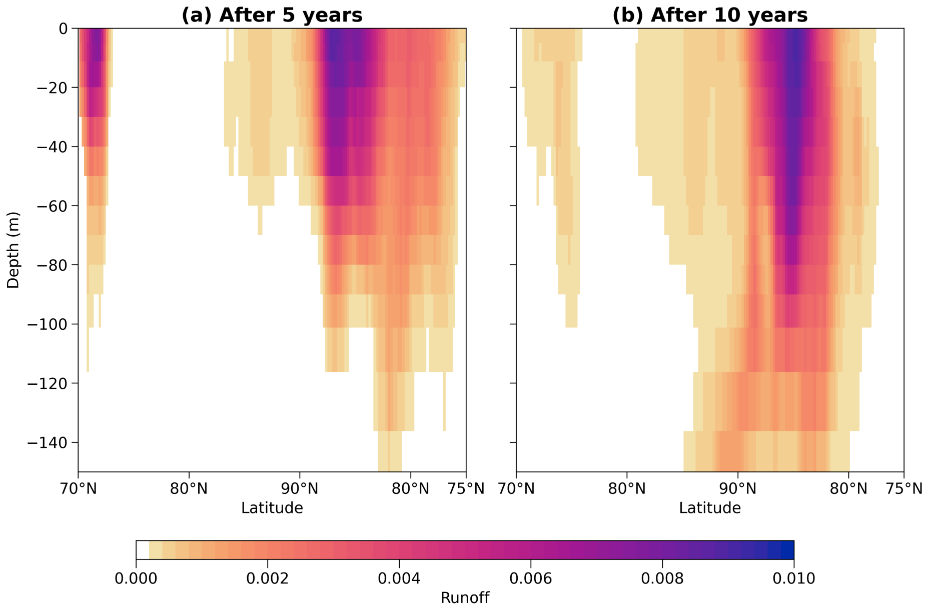

Supplementary Fig. 15: Passive tracers of river water in a transect crossing the central Arctic. a,b Total river water tracers in FESOM2 historical simulations: (**a**) five years after being released and (**b**) ten years after being released. The location of the transect is denoted in Supplementary Fig. 11a.

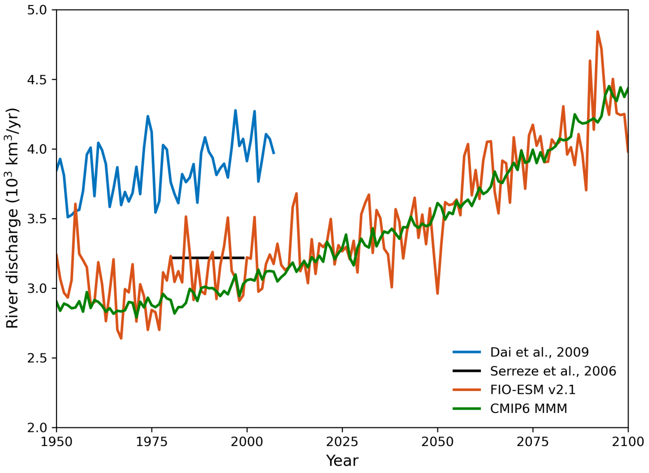

Supplementary Fig. 16: River runoff in the Arctic. The Arctic river runoff used in FESOM2 simulations (derived from FIO-ESM v2.1) compared with available reference estimates (Dai et al., 2009 and Serreze et al., 2006) and CMIP6 multi-model mean (MMM). The considered Arctic domain is enclosed by Fram Strait, Barents Sea Opening, Bering Strait and the northern boundary of the Canadian Arctic Archipelago. The future projection starting from 2015 corresponds to the SSP585 scenario.

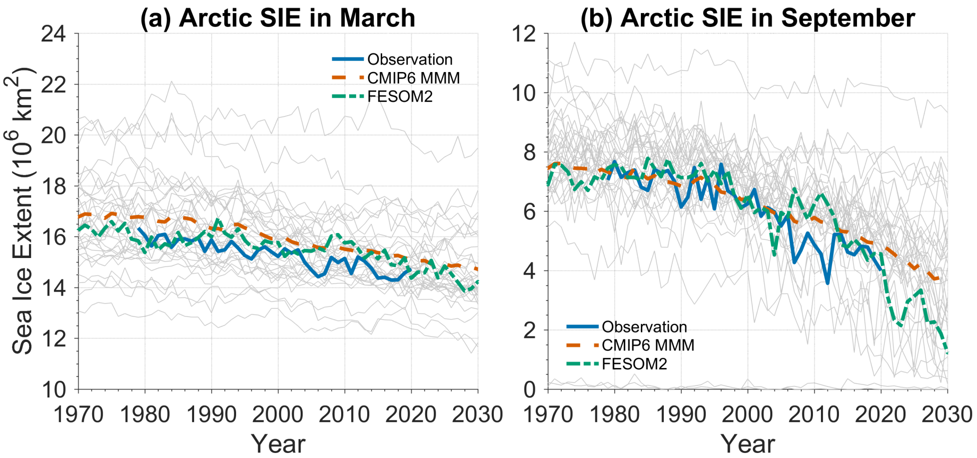

Supplementary Fig. 17: Arctic sea ice extent (SIE) in models and observations. Arctic SIE in (**a**) March and (**b**) September. The blue, red and green lines indicate satellite observations, CMIP6 multi-model mean (MMM), and FESOM2 simulation, respectively. Each thin gray line denote one CMIP6 model.
