# Supplementary material for: Warming transforms the western Arctic Ocean into a hub of drifting matter
Source: Nat Commun. 2026 Jun 16;17:5317. doi: 10.1038/s41467-026-74439-5 (PMC13272592; doi:10.1038/s41467-026-74439-5)
Supplement: Supplementary file 2 — Transparent Peer Review file [file 41467_2026_74439_MOESM2_ESM.pdf]

nature portfolio

Peer Review File

Warming transforms the western Arctic Ocean into a hub of drifting matter

Corresponding Author: Dr Qiang Wang

This file contains all reviewer reports in order by version, followed by all author rebuttals in order by version.

Version 1:

Reviewer comments:

Reviewer #1

(Remarks to the Author)
The model study by Wang and coworkers addresses in interesting and potentially important topic, namely the changed pathways of river water and associated suspended biogeochemical material in the Arctic Ocean - in a future climate compared to present-day conditions. Using an eddy-permitting ocean model and both Eulerian and Lagrangian calculations for river water transport, the authors find that a future reduced sea ice cover, with associated enhanced current and eddy energies, lead to both enhanced connectivity between different regions (eastern vs. western basins) of the Arctic and quicker exit of river water out of the Arctic. I must admit that these findings come at no great surprise (reduced sea ice cover-->more energetic currents-->enhanced advection and eddy stirring). But I found the manuscript to be quite well structured and written, and I agree that it could be published in Nature Communications.

As always, there are things that may be improved, both in terms of science and presentation. Below I offer a modest number of suggestions which could hopefully improve the manuscript some. I will address the authors directly. And even though I frame some of these comments as questions to the authors, I really ask that each be addressed not only to me but to the reader of a finished¨ paper.

My one general concern is that the story is somewhat lacking in dynamical explanations for the observed changes in flow path and dispersion:

1) You propose that the transpolar drift, the Beaufort gyre and shelfbreak jets all speed up in the future scenario. The explanation given is enhanced momentum transfer from the atmosphere due to a reduced sea ice cover.

a) In the case of the Beaufort gyre this seems reasonable. But does Fig. 2a,b really show a strengthening of the gyre (rather than strengthening of topographically locked boundary currents/shelf jets)? Would a plot of SSH for the two time periods show this better as it highlights larger scales - even if FWC is shown in a supplementary figure (see my comment about this below)?

b) That a reduced sea ice cover causes strengthened shelf edge jets is less obvious since these are, in part, connected to the circulation outside of the Arctic. You may want to mention this.

2) You appear to base the claim that the transpolar drift is also strengthening on Fig. 2a,b. What I see here is enhanced flow strength along the Lomonosov ridge. Is this, a narrow and topographically steered current, really the dynamical definition of the transpolar drift?

3) You claim that a strengthening of the Beaufort gyre causes more river water to enter the gyre, especially from Mackenzie river. But how is a strengthened gyre circulation actually dynamically tied to enhanced transport *through the gyre boundaries*? Are both related to enhanced surface Ekman convergence (a piling up of surface waters would lift sea surface, spinning up geostrophic currents)? If so, can this be shown in a figure of e.g. Ekman pumping velocities? Or is it enhanced eddy activity and eddy advection what does this? Or both? For this, see my comment below about a possible opposition between Ekman convergence and eddy (bolus) divergence in the Baufort.

Minor comments:

* It would be useful for the reader to be informed at an early stage that the ocean model used is eddy-permitting. This fact is now only mentioned in the Discussion and Methods sections. Also, I would like such a first mention of this to be accompanied with a brief discussion of what is meant by 'eddy-permitting'. Is it that the first internal deformation radius is resolved by a few grid cells? Note that the deformation radius is relevant specifically for the Eady model of baorclinic instability. As such, eddies (generated by Eady-instability) on the quite shallow shelves can be expected to be much less resolved. This is also to be expected over the continental slopes where modified Eady theory which accounts for a sloping bottom predicts smaller scales than what would be obtained over a flat bottom (e.g. Blumsack, S.L., Gierasch, P.J., 1972. Mars: the effects of topography on baroclinic instability. J. Atmos. Sci. 29, 1081–1089).

* A few words, early on, about how the atmospheric circulation is different in the future scenario would be useful.

Fig. 2 caption: "...ocean currents..." --> "...ocean current strength..." (since we only see magnitude, not direction).

l. 483-84. Is there a dynamical reason for why the accumulation of surface waters+suspended material in the Beaufort is higher than waters+material at depth? Can it be explained by enhanced surface Ekman convergence - an effect which does not reach very deep?

l. 491: "Due to the convergent nature of the Beaufort Gyre..." What is meant by this? The convergence of surface Ekman transport?

l. 574-77: How do enhanced eddy stirring and stronger Ekman convergence *work together* ("combine") to make the Beaufort Gyre a pan-Arctic convergence zone? Note, for example, that in simplified dynamical models of the BF gyre itself, eddy advection is thought to oppose the Ekman-driven cross-gyre flow (e.g. Manucharyan, G. E., & Spall, M. A. (2016). Wind-driven freshwater buildup and release in the Beaufort Gyre constrained by mesoscale eddies. Geophysical Research Letters, 43, 273–282).

Metods - Numerical model: FVCOM uses an unstructured grid. Was the resolution still fixed at 4.5 km in the Arctic and why?

l. 676-78: "...eddy-permitting...enabling the explicit simulation of mesoscale eddies." See comment above on what eddy-permitting really means. Maybe modify this sentence a bit, e.g. by saying that the largest eddies, generated by Eady-type instability, can be expected to be well-represented in deep waters. But also that the eddy field on the shelves and over continental slopes and ridge systems may not be well-resolved.

Methods - Particle tracking: Were the releases of tracers and particles done quantitatively similarly, e.g. with respect to seasonal variations?

l. 849-851: The claim that sea surface height is mainly determined by salinity (~density) distribution is interesting. Is this claim supported by reference [49]? Note that this implies that SSH variations are primarily baroclinic. The agreement between density and SSH is one-to-one if the bottom flow is zero, i.e. if there is perfect compensation. But bottom currents in the Arctic are generally not zero. So could you check to see if a plot of SSH looks like FWC, at least in the model where you should actually have access to the SSH field?

Reviewer #2

(Remarks to the Author)
Using numerical simulations based on FESOM2, this study investigate how future warming and changes in runoff may affect the transport and distribution of riverine discharge from the Siberian shelves and the MacKenzie River. The results show that the multi-scale ocean circulation changes associated with sea-ice decline (primarily, strengthened shelf-break jet, an intensified Transpolar Drift, and enhanced eddy activities) promote greater accumulation of materials in the western Arctic Ocean, increase cross-basin connectivity, and accelerate exports from the Arctic Ocean to subpolar regions. These findings have strong implications for Arctic-subarctic marine environments and the communities that depend on them.

The manuscript is well written, and its conclusions are well supported by the results presented. The study addressed an important and timely topic considering the rapid changes occurring in Arctic marine and terrestrial systems. I recommend acceptance of this manuscript for publication, pending minor revisions to address the questions and clarifications outlined below.

Key Discussion Points

I would like the authors to clarify one aspect of their modeling methodology. L 711-712 state that the tracer released is related to the amount of runoff. If so, does this imply that the total tracer input differs between the historical and future simulations? If yes, how does this difference in source magnitudes affect the comparison in absolute terms shown in Fig. 1?

Additional Suggestions/Clarifications

Fig. 1.: To help orient the readers, the river mouth could be indicated on the figure panels.

L191-192, “forced by the freshwater flux associated with river runoff”:
See the comment above regarding the absolute amount of tracer released. Where appropriate, please discuss how differences in source strength may affect the results.

L686, “climatological mean biases”:
Did you remove biases relative to a monthly or daily climatology? Does the timescale used to define the climatology differ among the various forcing variables?

L 711-712,”… equal to the respective river runoff”:
Again, see Key Discussion Points.

L743, “monthly release rate followed...”:
Could you clarify what is meant by “rate” in this context? The number of particles released appears to be fixed for each period (see lines 749-750, 1000 and 1350). Does the “release rate” refer to how quickly these fixed numbers of particles are released in time? Or is the total number of released particles scaled by runoff, or defined in some other way?

L908: do you mean supplemental Fig. 5g? There is no Fig. 5j.

L1510, supplemental Fig. 1:
The total number of particles released differs between historical and future scenarios, which suggests that the comparison may not be directly ‘apples to apples’. Could the authors clarify how this difference affects the interpretation of the results? In addition, why are there no particle shown in panel (a)? Have all particles exited the Arctic by that time?

Reviewer #3

(Remarks to the Author)
This paper examines the variability of thermohaline circulation in the Arctic in the context of global warming and river inflow. The findings are well-documented, although many doubts remain about the research methods.
1. Why were the 1985-2014 and 2071-2100 time periods chosen?
2. Why was only the SSP5-8.5 warming scenario examined?
3. Why is the ocean model's calculation period exactly 20 years, and why is the sensitivity of the results to this parameter not considered?
4. Why are other significant rivers in the Arctic basin not taken into account? Consider the Nelson, Churchill, Yana, Indigirka, Khatanga, Olenek, Pechora, and Northern Dvina rivers.
5. Was the ice area in the Arctic Ocean and its dynamics over time validated using modeling results? Ice processes have a significant effect on water circulation.
6. According to a number of papers, Earth system models do not accurately predict river runoff. This is also supported by the comparison data in this study. It would be far more reliable to model river discharge using hydrological models. It is critical to replicate not only the total flow of these Arctic rivers, but also the flow dynamics throughout time (interannual and seasonal). This is especially useful for major rivers in comparing the variability of river water influx into different Arctic seas. This will aid in the detailed verification of modern ocean currents as well as the assessment of future variability.
The authors are advised to significantly modify this paper.

Version 2:

Reviewer comments:

Reviewer #1

(Remarks to the Author)
I thank the authors for addressing all my comments. I now suggest publication, and only ask that the authors consider a couple of more questions/suggestions:

Streamlines added to Fig. 2a,b are not too effective in showing currents. Yes, they show direction, but the plotting routine's somewhat arbitrary choice in curves to draw reduces it's use as a quantitative measure. Since such streamlines don't give any information about the strength of currents (I realize that's that the color shows), consider instead contouring the SSH field (even though it's now in a supplementary figure).

Am not sure I understand the explanation behind the intensificatio of the shelfbreak jets. Lighter shelf waters sink less, thus intensifying the near-surface currents (shelfbreak jets)? So the mass (and FW) transport is just taking place nearer to the surface? Doesn't this fall to the side of the main story which is that enhanced momentum transfer to the ocean is the key

thing going on here? Please explain a bit more.

On a second reading I actually realize that I am unsure of what you mean by 'shelfbreak jets'. Please define this term better. Why the term 'jets'? Are they residing over the shelf break, meaning where the shelf connects to the continental slope, or are they over the slope itself? Are these jets associated with different dynamics than what is transporting Atlantic Water (at depth) cyclonically around the Arctic?

Reviewer #2

(Remarks to the Author)
The authors have adequately addressed my comments from the previous round of review. I have no further questions.

Reviewer #3

(Remarks to the Author)
The authors have provided largely convincing responses to my comments. However, I do recommend adding to the limitations of this study the fact that Earth system models simulate river runoff only very approximately, even for the largest rivers on the planet. At best, they can reproduce the multi-year mean annual runoff, but the reproduction of the seasonal cycle is extremely poor. It would be better to advise readers to rely on river runoff projections obtained from specialized hydrological models.

Open Access This Peer Review File is licensed under a Creative Commons Attribution 4.0 International License, which permits use, sharing, adaptation, distribution and reproduction in any medium or format, as long as you give appropriate credit to the original author(s) and the source, provide a link to the Creative Commons license, and indicate if changes were made.
In cases where reviewers are anonymous, credit should be given to 'Anonymous Referee' and the source.
The images or other third party material in this Peer Review File are included in the article’s Creative Commons license, unless indicated otherwise in a credit line to the material. If material is not included in the article’s Creative Commons license and your intended use is not permitted by statutory regulation or exceeds the permitted use, you will need to obtain permission directly from the copyright holder.
To view a copy of this license, visit https://creativecommons.org/licenses/by/4.0/

Reviewer #1

Dear reviewer,

Thank you very much for reviewing our paper and providing very helpful comments. We revised the paper following your comments. Please see our responses in detail below.

The model study by Wang and coworkers addresses an interesting and potentially important topic, namely the changed pathways of river water and associated suspended biogeochemical material in the Arctic Ocean - in a future climate compared to present-day conditions. Using an eddy-permitting ocean model and both Eulerian and Lagrangian calculations for river water transport, the authors find that a future reduced sea ice cover, with associated enhanced current and eddy energies, lead to both enhanced connectivity between different regions (eastern vs. western basins) of the Arctic and quicker exit of river water out of the Arctic. I must admit that these findings come at no great surprise (reduced sea ice cover-->more energetic currents-->enhanced advection and eddy stirring). But I found the manuscript to be quite well structured and written, and I agree that it could be published in Nature Communications.

Thank you for your recognition of our work and for your comments, which helped to improve the manuscript.

As always, there are things that may be improved, both in terms of science and presentation. Below I offer a modest number of suggestions which could hopefully improve the manuscript some. I will address the authors directly. And even though I frame some of these comments as questions to the authors, I really ask that each be addressed not only to me but to the reader of a finished paper.

My one general concern is that the story is somewhat lacking in dynamical explanations for the observed changes in flow path and dispersion:

1) You propose that the transpolar drift, the Beaufort gyre and shelfbreak jets all speed up in the future scenario. The explanation given is enhanced momentum transfer from the atmosphere due to a reduced sea ice cover.
a) In the case of the Beaufort gyre this seems reasonable. But does Fig. 2a,b really show a strengthening of the gyre (rather than strengthening of topographically locked boundary currents/shelf jets)? Would a plot of SSH for the two time periods show this better as it highlights larger scales - even if FWC is shown in a supplementary figure (see my comment about this below)?

We add the plots of SSH as you suggested. Together with the plots of FWC, they indicate the strengthening of the Beaufort Gyre (Supplementary Fig. 7). In addition, we add ocean current streamlines in the plots of ocean speeds, which can better show the locations of the main circulations (Fig. 2a,b).

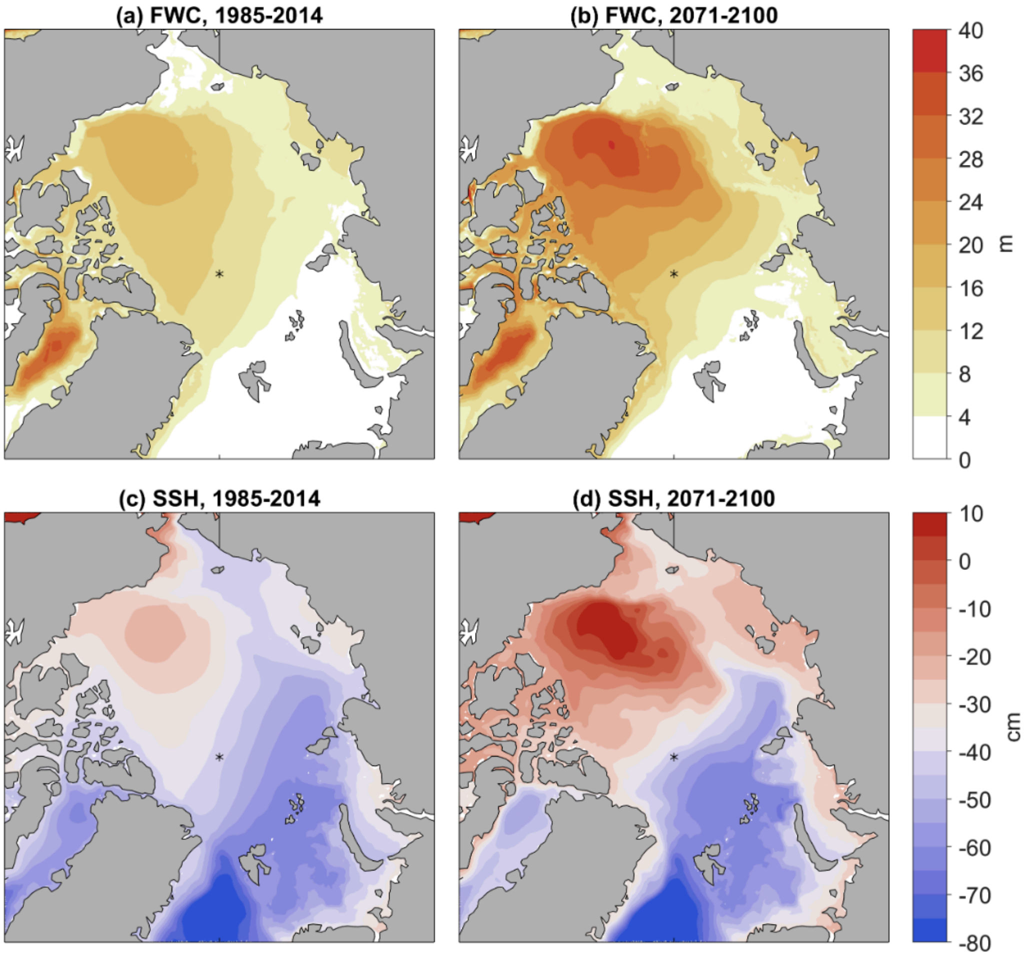

Supplementary Fig. 7: Freshwater content (FWC) and sea surface height (SSH) in different periods. a,b FWC averaged over (**a**) 1985–2014 and (**b**) 2071–2100. **c,d** The same as (**a,b**), but for SSH.

b) That a reduced sea ice cover causes strengthened shelf edge jets is less obvious since these are, in part, connected to the circulation outside of the Arctic. You may want to mention this.

A very good point! We did not find a significant increase in total Arctic inflow through Arctic gateways when we checked the net ocean volume transport. The main reason for the intensified shelfbreak jet along the Siberian slope is the vertical shift in the outflow from the continental shelf:

“Future warming and freshening over the continental shelf are expected to reduce shelf water density [51]. This reduction shifts the outflow from the continental shelf to shallower depths (Supplementary Fig. 8), thereby intensifying the shelfbreak jet along

the Siberian continental slope (Fig. 2a,b). The accelerated shelfbreak jet feeds into the Transpolar Drift, enhancing connectivity between the Siberian shelves and the subarctic seas, thereby reducing the timescale for Siberian river discharge to reach the central Arctic and Atlantic Ocean.” (Lines 544-556)

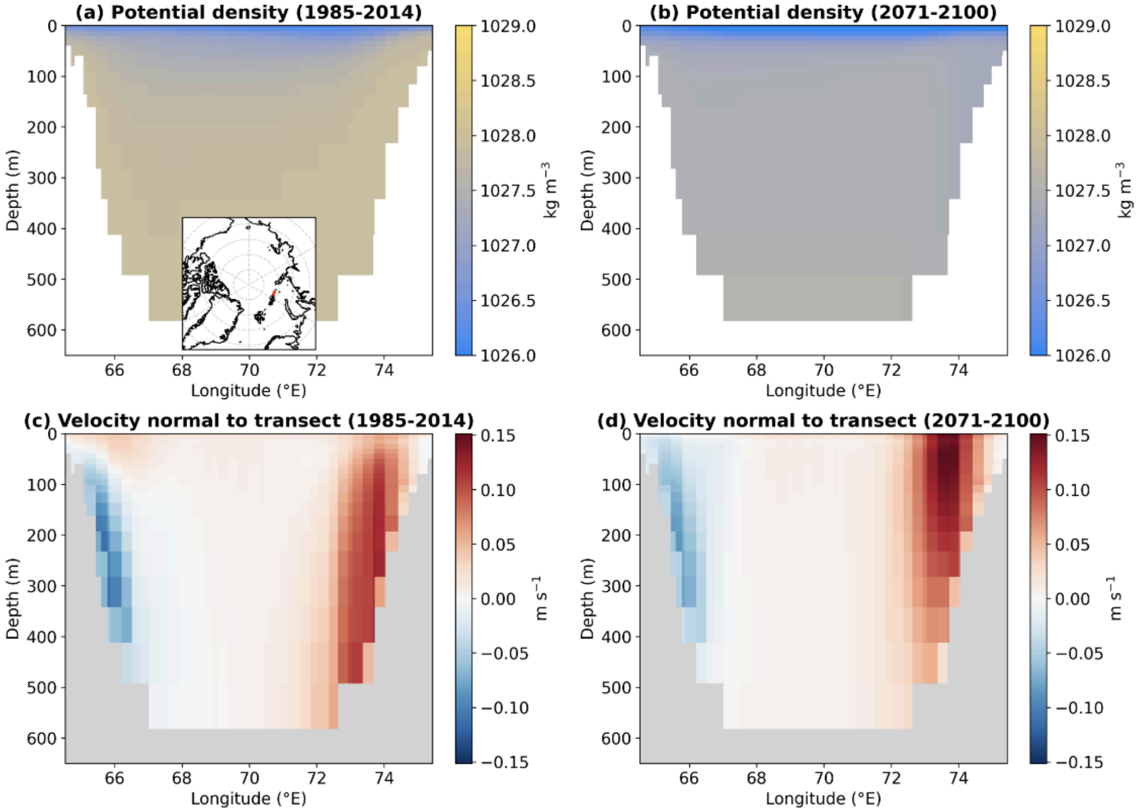

Supplementary Fig. 8: Decrease in density and associated upward shift of shelf water outflow in a warming climate. a,b Potential density across the St. Anna Trough in the (**a**) historical period and (**b**) future period. **c,d** The same as (**a,b**), but for velocity normal to the transect. Positive velocity indicates outflow from the shelf to the basin. The location of the transect is depicted in the inset in (**a**).

2) You appear to base the claim that the transpolar drift is also strengthening on Fig. 2a,b. What I see here is enhanced flow strength along the Lomonosov ridge. Is this, a narrow and topographically steered current, really the dynamical definition of the transpolar drift?

The plot of current speeds alone does not provide sufficient information. With the added SSH plots you suggested (Supplementary Fig. 7) and flow streamlines (Fig. 2a,b), the location and future strengthening of the Transpolar Drift can be better seen now.

3) You claim that a strengthening of the Beaufort gyre causes more river water to enter the gyre, especially from Mackenzie river. But how is a strengthened gyre circulation actually dynamically tied to enhanced transport *through the gyre boundaries*? Are both

related to enhanced surface Ekman convergence (a piling up of surface waters would lift sea surface, spinning up geostrophic currents)? If so, can this be shown in a figure of e.g. Ekman pumping velocities? Or is it enhanced eddy activity and eddy advection what does this? Or both? For this, see my comment below about a possible opposition between Ekman convergence and eddy (bolus) divergence in the Beaufort.

Our original sentence you referred to was misleading. There, we did not intend to address the counteracting effects of Ekman convergence and eddy fluxes, which is true. Instead, we were trying to recall a process mentioned in a paragraph prior to it: (Lines 222-280)

"For the Mackenzie River, part of its discharge enters the anticyclonic Beaufort Gyre circulation, while another portion exits the Arctic Ocean through the Canadian Arctic Archipelago straits (Fig. 1d). The partitioning between these two pathways depends on the strength of the anticyclonic gyre circulation. In a warming climate, consistent with the strengthening of the Beaufort Gyre (Fig. 2a,b), most of the Mackenzie River discharge follows the anticyclonic pathway and accumulates in the Canada Basin during the first five years (Fig. 1h)."

The sentence you referred to is revised now to avoid confusion: "The Mackenzie River discharge is projected to remain in the Arctic Ocean for a longer period in the future (Fig. 3j-l) …" (Line 368-369)

Minor comments:

* It would be useful for the reader to be informed at an early stage that the ocean model used is eddy-permitting. This fact is now only mentioned in the Discussion and Methods sections. Also, I would like such a first mention of this to be accompanied with a brief discussion of what is meant by 'eddy-permitting'. Is it that the first internal deformation radius is resolved by a few grid cells? Note that the deformation radius is relevant specifically for the Eady model of baorclinic instability. As such, eddies (generated by Eady-instability) on the quite shallow shelves can be expected to be much less resolved. This is also to be expected over the continental slopes where modified Eady theory which accounts for a sloping bottom predicts smaller scales than what would be obtained over a flat bottom (e.g. Blumsack, S.L., Gierasch, P.J., 1972. Mars: the effects of topography on baroclinic instability. J. Atmos. Sci. 29, 1081–1089).

We now first mention that the model is eddy-permitting in the last paragraph of the Introduction. In Methods, we then revised the text following your suggested expression in another comment:

"In this study, the horizontal resolution in the Arctic region is 4.5 km and is coarsened elsewhere to reduce computational cost (Supplementary Fig. 10a). The largest eddies, arising from baroclinic instability, are expected to be resolved in the deep basin, but not

over the shelves and along the continental slopes (Supplementary Fig. 10b,c).” (Lines 719-726)

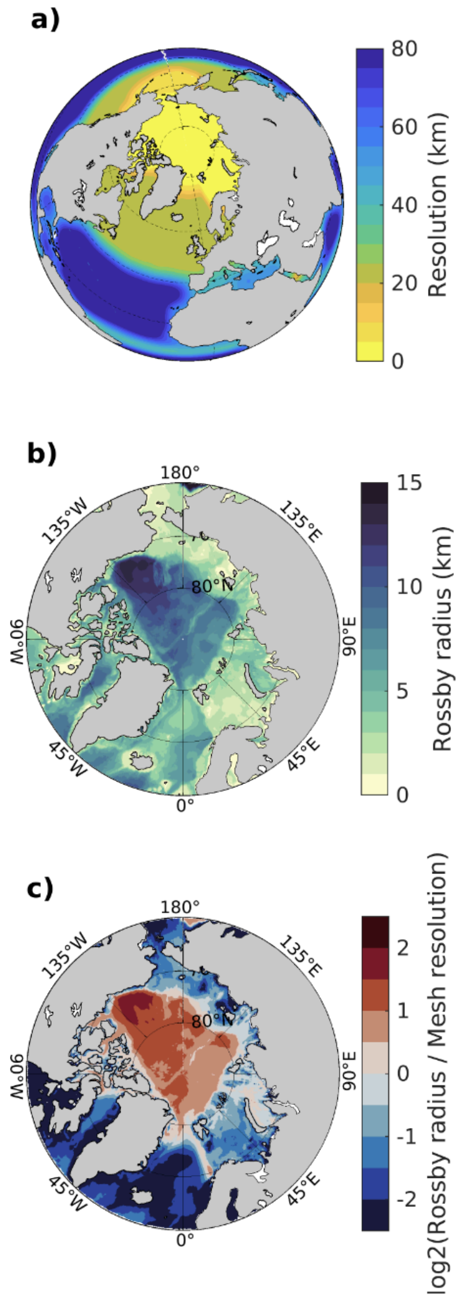

Supplementary Fig. 10: Model mesh resolution and first baroclinic Rossby radius. a Mesh resolution. **b** First baroclinic Rossby radius of deformation. **c** Ratio of first baroclinic Rossby radius to mesh resolution shown as $\log_2$ values. Eddy-permitting resolution requires the $\log_2$ value to be larger than zero.

* A few words, early on, about how the atmospheric circulation is different in the future scenario would be useful.

As the impact of sea ice decline through enhancing momentum transfer as suggested in the cited references is at least as important as changes in winds, we mention both aspects together at the beginning of the third result section. Discussing both in this mechanism section helps the narrative flow.
“Arctic sea ice is projected to decline substantially, while near-surface winds are expected to strengthen (Supplementary Fig. 6) …” (lines 537-539)

Fig. 2 caption: "...ocean currents..." --> "...ocean current strength..." (since we only see magnitude, not direction).
Flow streamlines are added to indicate directions.

l. 483-84. Is there a dynamical reason for why the accumulation of surface waters+suspended material in the Beaufort is higher than waters+material at depth? Can it be explained by enhanced surface Ekman convergence - an effect which does not reach very deep?
We agree. We revised the sentence: “This suggests that Siberian riverine materials floating near the surface have higher chances to be accumulated into the Canada Basin than those mixed to greater depths, likely because surface Ekman transport is largely restricted to the upper 10-20 m [52].” (lines 499-500)

l. 491: "Due to the convergent nature of the Beaufort Gyre..." What is meant by this? The convergence of surface Ekman transport?
The sentence is revised to “Due to surface Ekman convergenc …” (lines 506-507)

l. 574-77: How do enhanced eddy stirring and stronger Ekman convergence *work together* ("combine") to make the Beaufort Gyre a pan-Arctic convergence zone? Note, for example, that in simplified dynamical models of the BF gyre itself, eddy advection is thought to oppose the Ekman-driven cross-gyre flow (e.g. Manucharyan, G. E., & Spall, M. A. (2016). Wind-driven freshwater buildup and release in the Beaufort Gyre constrained by mesoscale eddies. Geophysical Research Letters, 43, 273–282).
The “eddy stirring” mentioned here refers to that across the Transpolar Drift, not in the Beaufort Gyre region. Waters dispersed from the Transpolar Drift into the Amerasian Basin can subsequently be influenced by anticyclonic winds over the Canada Basin.
We revised the sentence to avoid confusion: “The combined effects of enhanced eddy stirring across the Transpolar Drift and strengthened Ekman convergence over the Canada Basin …” Lines 619-621

Methods - Numerical model: FESOM uses an unstructured grid. Was the resolution still fixed at 4.5 km in the Arctic and why?

The functionality of the unstructured grid model is used to reduce resolution outside the Arctic Ocean. Higher resolution within the Arctic was not pursued in order to limit computational cost and enable sufficiently long simulations as we used.
"In this study, the horizontal resolution in the Arctic region is 4.5 km and is coarsened elsewhere to reduce computational cost (Supplementary Fig. 10a). The largest eddies, arising from baroclinic instability, are expected to be resolved in the Arctic deep basin, but not over the shelves and along the continental slopes (Supplementary Fig. 10b,c). The chosen resolution represents a compromise between our requirements and computational constraints." (lines 719-728)

l. 676-78: "...eddy-permitting...enabling the explicit simulation of mesoscale eddies." See comment above on what eddy-permitting really means. Maybe modify this sentence a bit, e.g. by saying that the largest eddies, generated by Eady-type instability, can be expected to be well-represented in deep waters. But also that the eddy field on the shelves and over continental slopes and ridge systems may not be well-resolved.
We revised the text following your suggestions. See our reply above.

Methods - Particle tracking: Were the releases of tracers and particles done quantitatively similarly, e.g. with respect to seasonal variations?
Yes. "The number of particles released in each month is scaled according to the seasonal variability of river runoff." (Line 802-803) However, online tracers are conservative quantities, exactly equal to runoff input to the ocean, while for offline particles it only makes sense to interpret their fractions for quantities, as we did in the paper. Overall, the two methods complement each other, and consistently support our main findings.

l. 849-851: The claim that sea surface height is mainly determined by salinity (~density) distribution is interesting. Is this claim supported by reference [49]? Note that this implies that SSH variations are primarily baroclinic. The agreement between density and SSH is one-to-one if the bottom flow is zero, i.e. if there is perfect compensation. But bottom currents in the Arctic are generally not zero. So could you check to see if a plot of SSH looks like FWC, at least in the model where you should actually have access to the SSH field?
In Supplementary Fig. 7 shown above, SSH and FWC patterns consistently depict changes in the gyre and Transpolar Drift.

Reviewer #2

Dear reviewer,

Thank you very much for reviewing our paper and providing very helpful comments. We revised the paper following your comments. Please see our responses in detail below.

Using numerical simulations based on FESOM2, this study investigates how future warming and changes in runoff may affect the transport and distribution of riverine discharge from the Siberian shelves and the MacKenzie River. The results show that the multi-scale ocean circulation changes associated with sea-ice decline (primarily, strengthened shelf-break jet, an intensified Transpolar Drift, and enhanced eddy activities) promote greater accumulation of materials in the western Arctic Ocean, increase cross-basin connectivity, and accelerate exports from the Arctic Ocean to subpolar regions. These findings have strong implications for Arctic-subarctic marine environments and the communities that depend on them.

The manuscript is well written, and its conclusions are well supported by the results presented. The study addressed an important and timely topic considering the rapid changes occurring in Arctic marine and terrestrial systems. I recommend acceptance of this manuscript for publication, pending minor revisions to address the questions and clarifications outlined below.

Thank you for your recognition of our work and for your comments, which helped to improve our manuscript.

Key Discussion Points

I would like the authors to clarify one aspect of their modeling methodology. L 711-712 state that the tracer released is related to the amount of runoff. If so, does this imply that the total tracer input differs between the historical and future simulations? If yes, how does this difference in source magnitudes affect the comparison in absolute terms shown in Fig. 1?
To answer your question, we added particle tracking experiments using 1000 particles in the future scenario. These offline experiments are relatively easy to do compared with repeating the numerical model simulations. They can tell us ocean circulation changes are the main driver for the difference between results under different climate conditions:

“The absolute values of river water content shown in Fig. 1 are influenced by both ocean circulation and the amount of river discharge, which increases under a warming

climate. By comparing particle tracking experiments in which the number of particles either accounts for the increase in river runoff or remains unchanged (see Methods), we find that the quantitative differences between these experiments are much smaller than those between the two periods considered (Supplementary Figs. 1 and 2). This suggests that changes in ocean circulation mainly drive the differences in the spatial distribution of river waters, particularly their faster dispersal under future climate." (lines 282-294)

"We performed sensitivity experiments using 1000 particles in the future scenario. The resulting spatial distribution and the fraction of particles in the Canada Basin are comparable to those obtained with 1350 particles (Supplementary Figs. 2 and 12). The differences between these experiments (Supplementary Figs. 2 and 12) are much smaller than the climate change signal (Fig. 4c-f and Supplementary Fig. 1)." (820-831)

To support our main findings, we showed both absolute volume and the fractions relative to the total tracer/particles (e.g., Fig. 3 and Fig. 4b) in the paper. The fractions are not influenced by the total amount.

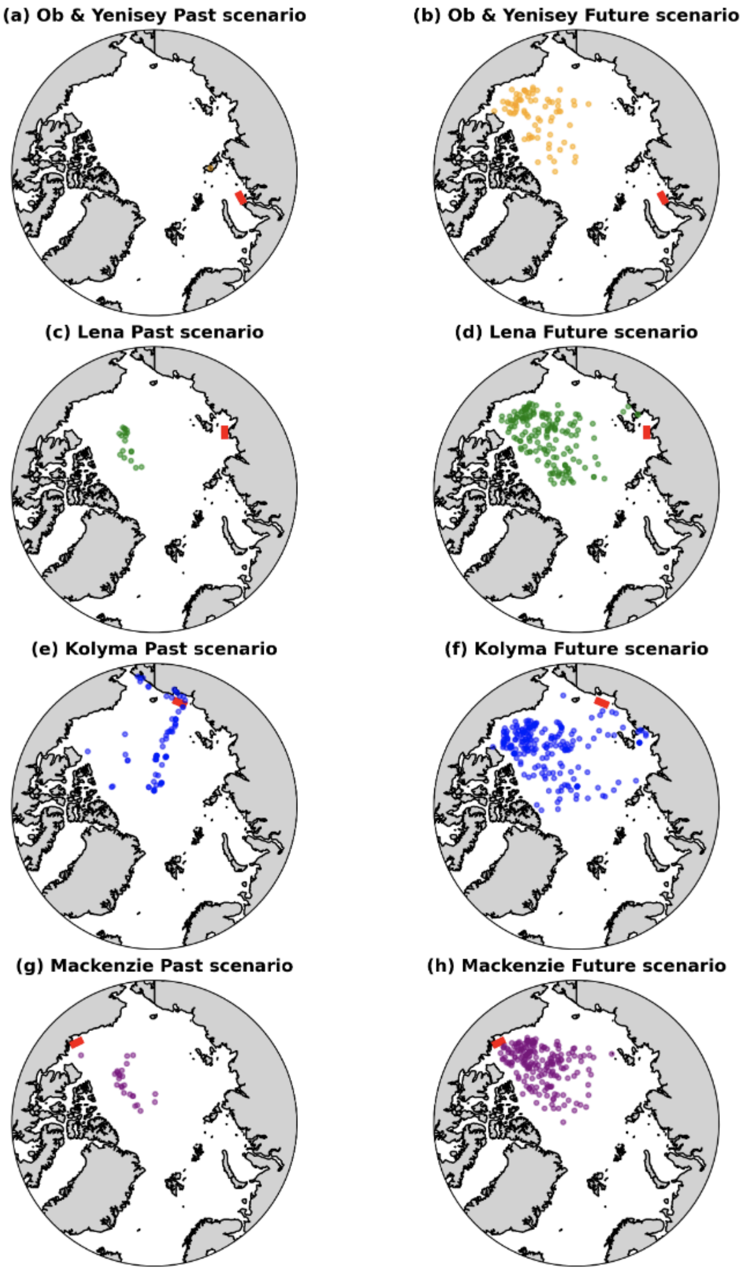

Supplementary Fig. 1: Spatial distribution of surface particles after being released for 20 years for the four major river systems. a,b Particles released from Ob–Yenisey rivers in the (**a**) historical and (**b**) future periods. **c,d** The same as (**a,b**), but for the Lena River. **e,f** The same as (**a,b**), but for the Kolyma River. **g,h** The same as (**a,b**), but for the Mackenzie River. The results from the first experiment ensemble member are shown, in which the particles are released in 1985 (**a,c,e,g**) and 2071 (**b,d,f,h**). Only every fourth particles are shown for a better illustration. The locations of particle release are indicated by red bars. The absence of particles in (**a**) indicates that all particles have exited the Arctic.

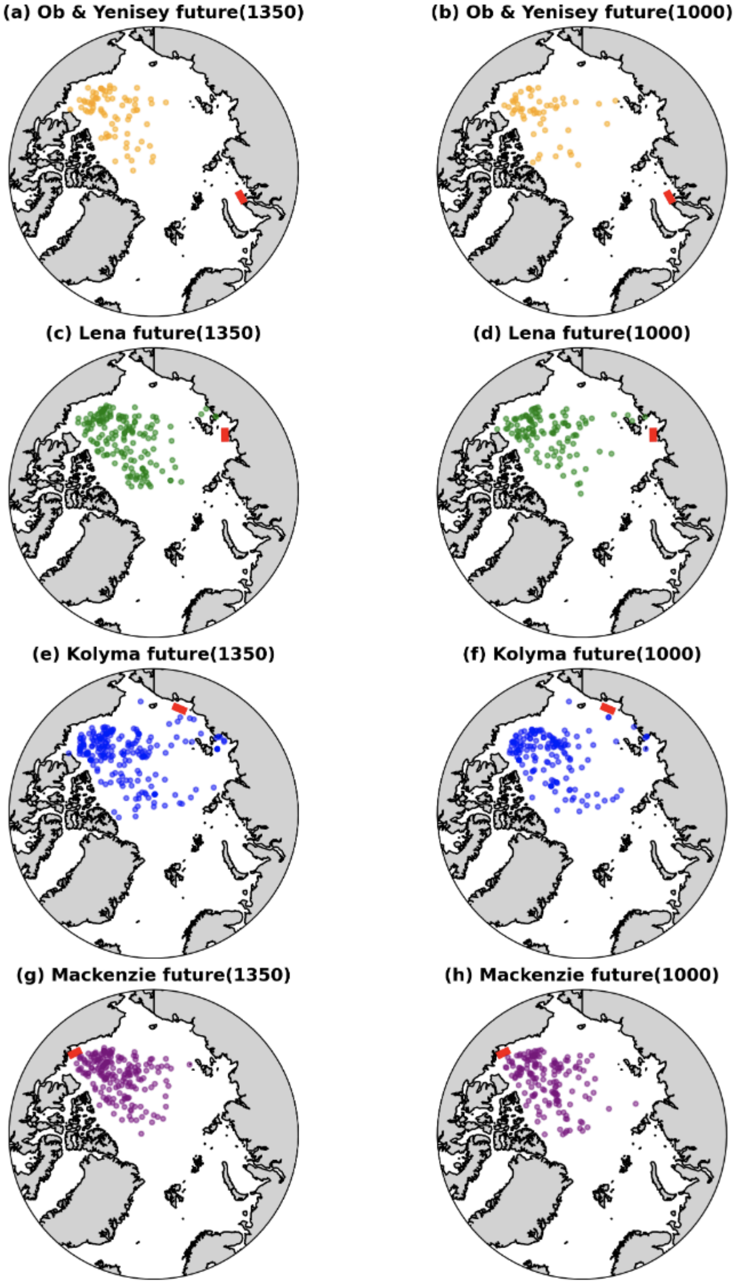

Supplementary Fig. 2: Spatial distribution of surface particles in experiments with different number of released particles. a,b Particles after being released for 20 years from Ob–Yenisey rivers in the experiment with (**a**) 1350 particles and (**b**) 1000 particles. The particles are released in 2071. **c,d** The same as (**a,b**), but for the Lena River. **e,f** The same as (**a,b**), but for the Kolyma River. **g,h** The same as (**a,b**), but for the Mackenzie River. Only every fourth particles are shown for a better illustration. The locations of particle release are indicated by red bars.

Additional Suggestions/Clarifications

Fig. 1.: To help orient the readers, the river mouth could be indicated on the figure panels.
done

L191-192, “forced by the freshwater flux associated with river runoff”:
See the comment above regarding the absolute amount of tracer released. Where appropriate, please discuss how differences in source strength may affect the results.
See our reply above.

L686, “climatological mean biases”:
Did you remove biases relative to a monthly or daily climatology? Does the timescale used to define the climatology differ among the various forcing variables?
We revise the text to make it clear: “To reduce the impacts of systematic biases in the atmospheric forcing on the FESOM2 simulations, climatological 3-hourly mean biases relative to atmospheric reanalysis data were subtracted from the forcing fields, including near-surface winds and air temperature” (lines 736-741)

L 711-712,”… equal to the respective river runoff”: Again, see Key Discussion Points.
See our reply above.

L743, “monthly release rate followed...”:
Could you clarify what is meant by “rate” in this context? The number of particles released appears to be fixed for each period (see lines 749-750, 1000 and 1350). Does the “release rate” refer to how quickly these fixed numbers of particles are released in time? Or is the total number of released particles scaled by runoff, or defined in some other way?
We revised the text to make this clear: “The number of particles released in each month is scaled according to the seasonal variability of river runoff.” (lines 802-803)

L908: do you mean supplemental Fig. 5g? There is no Fig. 5j.
corrected.

L1510, supplemental Fig. 1: The total number of particles released differs between historical and future scenarios, which suggests that the comparison may not be directly ‘apples to apples’. Could the authors clarify how this difference affects the interpretation of the results? In addition, why are there no particles shown in panel (a)? Have all particles exited the Arctic by that time?

As replied to the major comment above, we added sensitivity experiments to illustrate the difference. And a plot of no-particles means all particles have exited the Arctic by that time (we mention this in the figure caption now).

Reviewer #3

Dear reviewer,

Thank you very much for reviewing our paper and providing very helpful comments. We revised the paper following your comments. Please see our responses in detail below.

This paper examines the variability of thermohaline circulation in the Arctic in the context of global warming and river inflow. The findings are well-documented, although many doubts remain about the research methods.
Thank you for your recognition of our work and for your comments, which helped to improve our manuscript.

1. Why were the 1985-2014 and 2071-2100 time periods chosen?
These are the two periods usually taken to represent the present-day and long-term-future climate conditions in CMIP6 studies and IPCC report. The CMIP6 historical simulations cover the historical period till 2014, so one takes the last 20 or 30 years before 2014 to represent the present-day (recent past) baseline. The end-of-century (the last 20 or 30 years of this century) condition is considered a long-term future. We used 30 years because we did 11 particle tracking experiments, each lasting 20 years, spanning 30 years in total.

2. Why was only the SSP5-8.5 warming scenario examined?
During the revision, we added particle tracking experiments for the SSP245 warming scenario to show the sensitivity to warming levels.

“A future projection simulation with FESOM2 was also conducted under the CMIP6 SSP245 warming scenario. In contrast to SSP585, which is the upper-bound emission scenario often described as ``fossil-fueled development'' with no climate mitigation, the SSP245 scenario represents a ``middle of the road'' future with moderate climate protection measures. This simulation is identical to the SSP585 simulation described above, except for the scenario forcing. Although river runoff tracers are not available in this simulation, particle tracking experiments were conducted in the same manner as for the SSP585 scenario. A comparison between the two scenarios shows that stronger warming leads to a greater accumulation of Siberian river particles in the Canada Basin (Supplementary Fig. 5)..” (Lines 864-880)

“To assess the robustness of our findings, we also conducted particle tracking experiments for the CMIP6 SSP245 scenario. In both SSP245 and SSP585 scenarios, the particles released from the Siberian rivers show clearly increased accumulation in

the Canada Basin relative to the historical period, with the warmer SSP585 scenario leading to even greater accumulation of Siberian particles (Supplementary Fig. 5).” (Lines 519-526)

“The presence of Siberian river particles in the Canada Basin increases from the SSP245 to the warmer SSP585 scenario, whereas the accumulation of Mackenzie River particles is similar between the two scenarios (Supplementary Fig. 5). The continued increase in the contribution of Siberian river discharge to the Beaufort Gyre under stronger warming, in contrast to the saturated local contribution from the Mackenzie River, highlights the growing importance of enhanced eddy stirring across the Transpolar Drift.” (Lines 593-604)

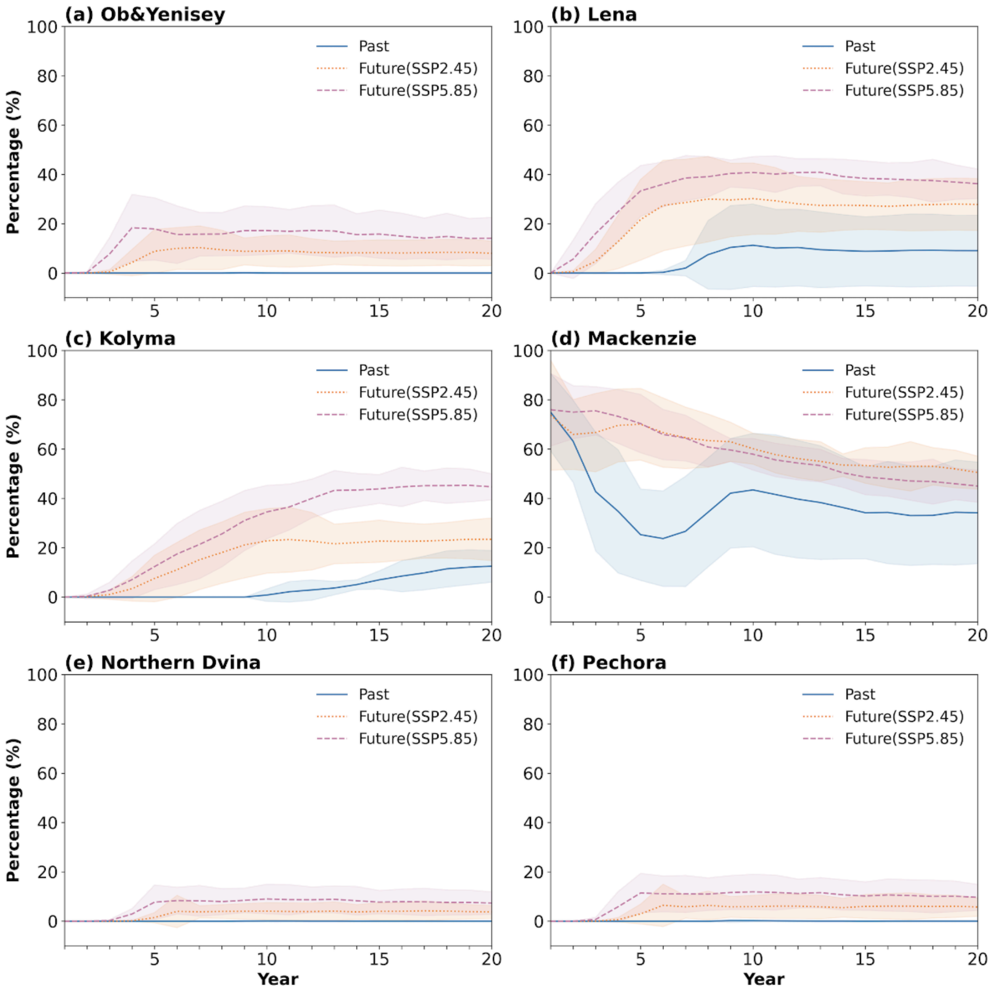

Supplementary Fig. 5: Proportion of surface particles in the Canada Basin relative to the total released particles in different CMIP6 scenarios. a-f Fraction of surface particles in the Canada Basin released from (**a**) Ob–Yenisey, (**b**) Lena, (**c**) Kolyma, (**d**) Mackenzie, (**e**) Northern Dvina, and (**f**) Pechora. The shading areas indicate one standard deviation of 11 particle tracking experiments.

3. Why is the ocean model's calculation period exactly 20 years, and why is the sensitivity of the results to this parameter not considered?

The ocean model calculation covers 200 years, from 1900 to 2100. For our purpose, just the tracers were added in the simulation for the chosen 20-year periods within the two considered representative periods (present day and long-term future). We investigated the time evolution during these periods, not only the final tracer distribution. In addition, we have a couple of ensembles of 11 particle tracking experiments, which allow us to show internal variability. This information can be considered as sensitivity for understanding internal variability. Overall, the results of online tracers and offline particles complement each other, consistently supporting our main conclusions.

4. Why are other significant rivers in the Arctic basin not taken into account? Consider the Nelson, Churchill, Yana, Indigirka, Khatanga, Olenek, Pechora, and Northern Dvina rivers.

In our tracer simulations, we took into account all discharge in each corresponding shelf sea. We revised the text in Methods to make it clear:

"To investigate the circulation of Arctic river discharge, we introduced passive tracers to represent river runoff in our model simulations. We focused on the four largest Arctic river systems: the Ob-Yenisey (Kara Sea), Lena (Laptev Sea), Kolyma (East Siberian Sea), and Mackenzie (Beaufort Sea), assigning one passive tracer to each. For each shelf sea, all river discharge was included in the tracer associated with the representative major river. As discharge from these major rivers dominates over that from other rivers, each tracer is referred to by the corresponding major river, although it represents the total runoff from the entire shelf sea." (lines 754-765)

For offline particle tracking experiments, we have to decide the location of particle release. In this case, we just took the river mouth of the major river from each shelf sea to conduct particle tracking experiments. They can represent the overall change in each corresponding shelf sea, because they are much larger than any other individual rivers, even the "next eight largest":

"*There are six Arctic rivers in this pan-Arctic watershed that have basin area's exceeding 500000km2 (the Ob', Yenisey, Lena, Mackenzie, Yukon and Kolyma). Combined these "Big 6" cover 67% of the pan-Arctic watershed and 63% of the total discharge into the Arctic ocean. The next eight largest rivers and their watersheds together only cover an additional 11% of this area and 16% of the discharge, with 22% of the area and 21% of the discharge left for the remaining 'smaller rivers' of the Arctic.*" (https://emodnet.ec.europa.eu/en/river-inputs-0)

Note that Yukon discharges southward into the Bering Sea, outside the domain studied in this paper, so we excluded it.

Our method is consistent with the way we present the results of the particle tracking experiments: we only discuss the fraction of particles relative to the total number released:

“Because the concentrations of drifting materials in river runoff are unknown and vary with material type, river, and future changes, particle distributions in the ocean were analyzed in terms of fractions, that is, the fraction of particles entering the Canada Basin relative to the total number released from each river. In this approach, the absolute number of released particles does not affect the results as long as the sample size is sufficiently large. We performed sensitivity experiments using 1000 particles in the future scenario. The resulting spatial distribution and the fraction of particles in the Canada Basin are comparable to those obtained with 1350 particles (Supplementary Figs. 2 and 12). The differences between these experiments (Supplementary Figs. 2 and 12) are much smaller than the climate change signal (Fig. 4c-f and Supplementary Fig. 1).” (lines 812-831)

During the revision, we added particle tracking experiments for the two rivers in the Barents Sea, as this marginal sea was missing in our previous analysis:

“The four major river systems considered above represent the four primary Arctic shelf seas but do not include the Barents Sea. Although discharge from the two largest rivers in the Barents Sea sector (the Pechora and the Northern Dvina via the White Sea) is substantially smaller than that of the four major Arctic rivers, we conducted particle tracking experiments for these rivers to assess the future evolution of Barents Sea discharge pathways. With these additional experiments, we can get an overview of all shelf seas inside the Arctic domain (enclosed by Fram Strait, Barents Sea Opening, Bering Strait and the northern boundary of the Canadian Arctic Archipelago). By comparing the fraction of particles entering the Canada Basin relative to the total number released, we can reveal how drifting matter from different shelf seas is differently influenced by climate change.” (Lines 846-862)
“Particles representing the river discharge in the Barents Sea also show increased accumulation in the Canada Basin under climate warming (Supplementary Fig. 4), although the corresponding fractions are lower than those of the four major river systems (Supplementary Fig. 5).” (Lines 512-517)

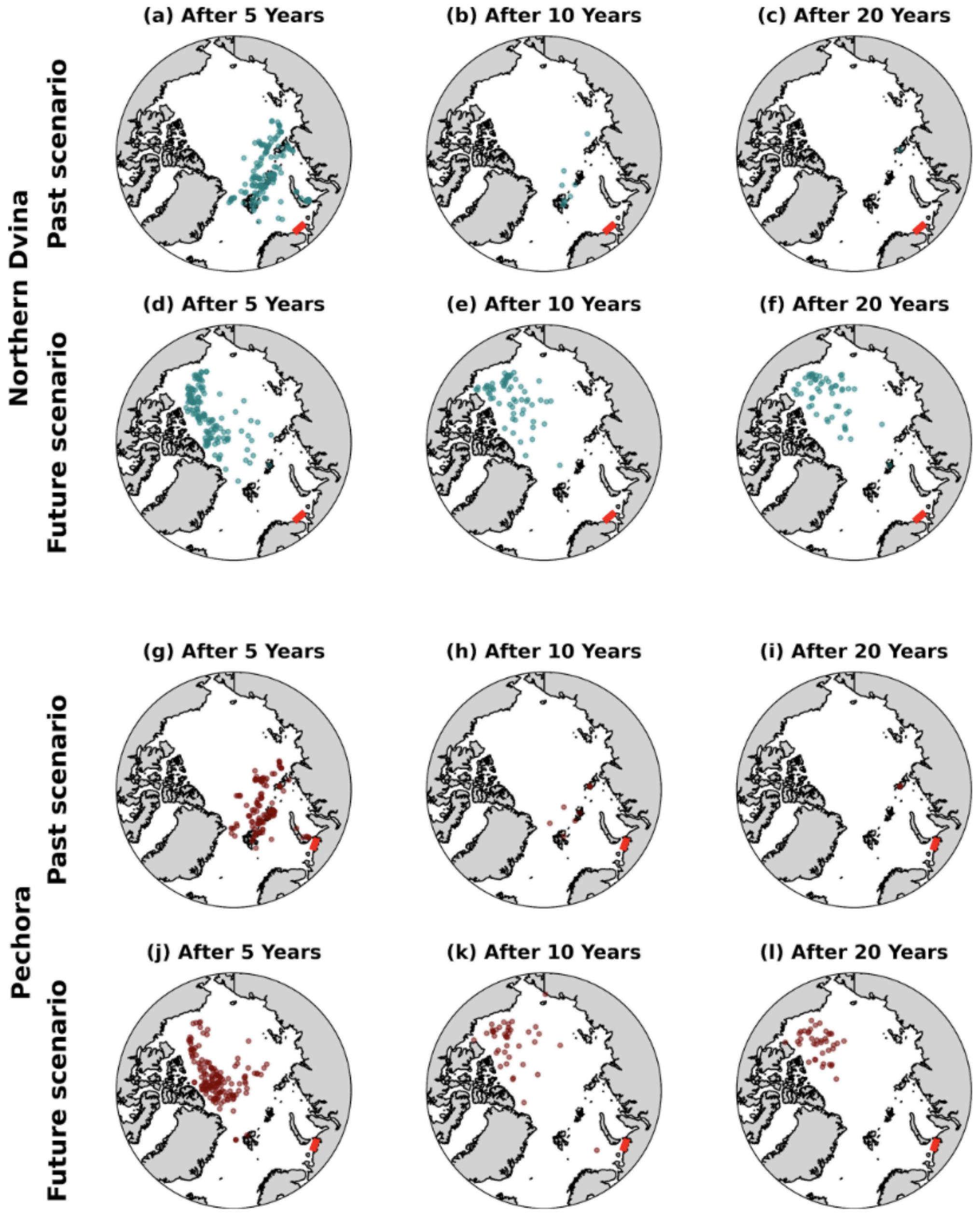

Supplementary Fig. 4: Spatial distribution of surface particles for the Barents Sea rivers. a-c Particles released from Northern Dvina in the historical period: after (**a**) 5 years, (**b**) 10 years, and (**c**) 20 years. **d-f** The same as (**a-c**), but for the future period. **g-l** The same as (**a-f**), but for the Pechora River. Only every fourth particles are shown for a better illustration. The locations of particle release are indicated by red bars. Northern Dvina particles are released at the White Sea exit. An absence of particles indicates that all particles have exited the Arctic at that time.

5. Was the ice area in the Arctic Ocean and its dynamics over time validated using modeling results? Ice processes have a significant effect on water circulation.

We added a figure (Supplementary Fig. 17) to show the time series of sea ice extent. By comparing our simulation to the time evolution of observations and CMIP6 models, we show that our simulation has a relatively good performance in representing sea ice (lines 1051-1057):

"In the historical period, FESOM2 adequately reproduces both the satellite-observed magnitudes and declining trends in Arctic summer and winter sea ice extent (Supplementary Fig. 17). The coarse-resolution CMIP6 models show large inter-model spreads in both magnitudes and trends."

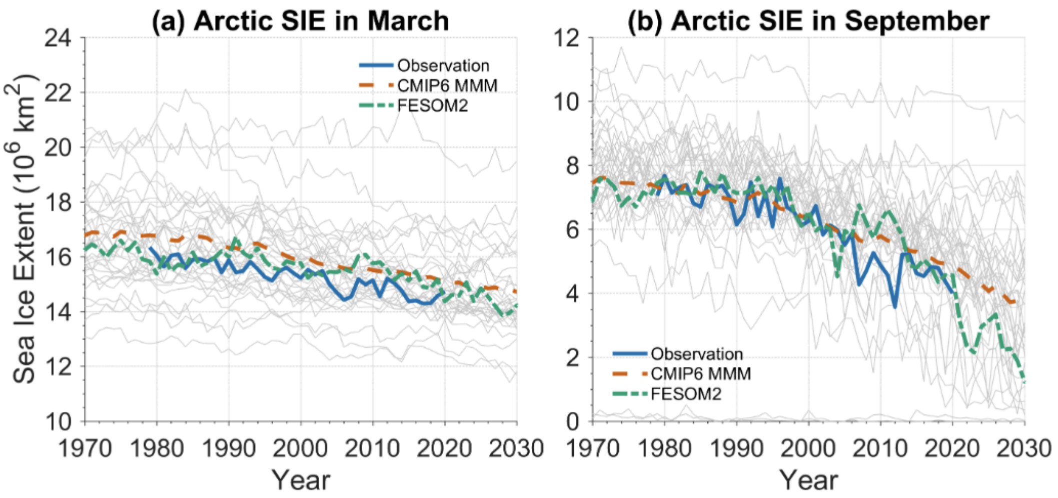

Supplementary Fig. 17: Arctic sea ice extent (SIE) in models and observations. Arctic SIE in (**a**) March and (**b**) September. The blue, red and green lines indicate satellite observations, CMIP6 multi-model mean (MMM), and FESOM2 simulation, respectively. Each thin gray line denote one CMIP6 model.

6. According to a number of papers, Earth system models do not accurately predict river runoff. This is also supported by the comparison data in this study. It would be far more reliable to model river discharge using hydrological models. It is critical to replicate not only the total flow of these Arctic rivers, but also the flow dynamics throughout time (interannual and seasonal). This is especially useful for major rivers in comparing the variability of river water influx into different Arctic seas. This will aid in the detailed verification of modern ocean currents as well as the assessment of future variability.
The authors are advised to significantly modify this paper.

Our evaluation of Arctic river runoff indicates that the runoff forcing used to drive the FESOM2 simulations is reliable, as it is actually comparable to the climatological estimates of Serreze et al. (2006) for the historical period. Furthermore, climate models do simulate river runoff using coupled hydrological components (i.e., land surface models combined with river routing schemes) that interact with the atmosphere and the broader climate system. Our understanding of future river runoff changes can only rely

on climate model projections. The projected evolution of runoff that we used is also consistent with the CMIP6 multi-model mean, further supporting its adequacy. Moreover, owing to limited observations, uncertainties across different reference estimates remain substantial. We revised the text to better explain and avoid confusion:

“During the historical period, the FIO-ESM v2.1 runoff is comparable to the observation-based climatological estimate [78], while being lower than another estimate combining observations and model simulations [79] (Supplementary Fig. 16). It is also very close to the CMIP6 multi-model mean in terms of both magnitude and future evolution. We note that available reference estimates of Arctic river runoff are subject to substantial uncertainty due to sparse gauging networks and the need to fill ungauged regions and periods using extrapolation and model simulations [78, 79]”

(Lines 1040-1050)

Reviewer #1

Dear reviewer,

Thank you very much for reviewing our paper again. We revised the paper following your comments.

I thank the authors for addressing all my comments. I now suggest publication, and only ask that the authors consider a couple of more questions/suggestions:

Streamlines added to Fig. 2a,b are not too effective in showing currents. Yes, they show direction, but the plotting routine's somewhat arbitrary choice in curves to draw reduces it's use as a quantitative measure. Since such streamlines don't give any information about the strength of currents (I realize that's that the color shows), consider instead contouring the SSH field (even though it's now in a supplementary figure).
We now show SSH contour lines instead of flow streamlines in Fig. 2a,b as you suggested.

Am not sure I understand the explanation behind the intensification of the shelfbreak jets. Lighter shelf waters sink less, thus intensifying the near-surface currents (shelfbreak jets)? So the mass (and FW) transport is just taking place nearer to the surface? Doesn't this fall to the side of the main story which is that enhanced momentum transfer to the ocean is the key thing going on here? Please explain a bit more.
The effect of the reduction in near-surface density is an important finding after your previous comment. That is, sea ice decline enhances momentum transfer (thus contributing to stronger Beaufort Gyre), but the reduction in surface density intensifies the shelfbreak current along the Siberian continental slope. In the mechanism section, we separate the first paragraph into two paragraphs, with each discussing one mechanism. This makes it clearer that both the two mechanisms are important in driving the changes in ocean circulation. The second paragraph addresses the second mechanism:

"Future warming and freshening over the Siberian shelves are expected to reduce shelf-water density [51]. This reduction shifts the ocean current toward shallower depths, as indicated by the velocity of the outflow from the shelves into the Eurasian Basin (Supplementary Fig. 8). As a result, the shelfbreak current along the upper continental slope intensifies and feeds an accelerated Transpolar Drift (Fig. 2a,b). As the upper branch of the Arctic Circumpolar Boundary Current, the shelfbreak current plays an important role in transporting upper-ocean water masses [53]. We find that, under future warming, the accelerated shelfbreak current and Transpolar Drift enhance upper-ocean connectivity between the Siberian shelves and the subarctic seas, reducing the timescale for Siberian river discharge to reach the central Arctic and the North Atlantic Ocean (Figs. 1 and 3) " (Lines 374-390)

On a second reading I actually realize that I am unsure of what you mean by 'shelfbreak jets'. Please define this term better. Why the term 'jets'? Are they residing over the shelf break, meaning where the shelf connects to the continental slope, or are they over the slope itself? Are these jets associated with different dynamics than what is transporting Atlantic Water (at depth) cyclonically around the Arctic?
We changed "shelfbreak jet" to "shelfbreak current" to be consistent with previous studies. Shelfbreak current is the upper branch of the Arctic Circumpolar Boundary Current, located over the upper continental slope close to the shelfbreak. The current was defined and described in previous studies (e.g, in ref 53). We explained it with citing the reference now (see the text quoted in the reply above). Atlantic Water boundary current is located at deeper depth, being another branch of the Arctic Circumpolar Boundary Current.

Reviewer #3

The authors have provided largely convincing responses to my comments. However, I do recommend adding to the limitations of this study the fact that Earth system models simulate river runoff only very approximately, even for the largest rivers on the planet. At best, they can reproduce the multi-year mean annual runoff, but the reproduction of the seasonal cycle is extremely poor. It would be better to advise readers to rely on river runoff projections obtained from specialized hydrological models.

Dear reviewer,

Thank you very much for reviewing our paper again. We added this comment at the end of the paper:

"In addition, simulations of river runoff in climate models have large uncertainties [67], which emphasizes the need for improved runoff projections to better constrain future changes and their climatic and ecological impacts." (Lines 541-545)
